# Surface-Exposed Protein Moieties of *Burkholderia cenocepacia* J2315 in Microaerophilic and Aerobic Conditions

**DOI:** 10.3390/vaccines12040398

**Published:** 2024-04-09

**Authors:** António M. M. Seixas, Carolina Silva, Joana M. M. Marques, Patrícia Mateus, Manuel J. Rodríguez-Ortega, Joana R. Feliciano, Jorge H. Leitão, Sílvia A. Sousa

**Affiliations:** 1 Department of Bioengineering, IBB—Institute for Bioengineering and Biosciences, Instituto Superior Técnico, Universidade de Lisboa, Av. Rovisco Pais, 1049-001 Lisboa, Portugal; antonio.seixas@tecnico.ulisboa.pt (A.M.M.S.); joanammmarques@tecnico.ulisboa.pt (J.M.M.M.); patricia.mateus@tecnico.ulisboa.pt (P.M.); joana.feliciano@tecnico.ulisboa.pt (J.R.F.); 2 Associate Laboratory, i4HB—Institute for Health and Bioeconomy, Instituto Superior Técnico, Universidade de Lisboa, Av. Rovisco Pais, 1049-001 Lisboa, Portugal; 3 Departamento de Bioquímica y Biología Molecular, Universidad de Córdoba, Campus de Excelencia Internacional CeiA3, 14071 Córdoba, Spain; mjrodriguez@uco.es

**Keywords:** *Burkholderia cenocepacia*, cystic fibrosis, surfomics, microaerophilic conditions, aerobic conditions

## Abstract

*Burkholderia cepacia* complex infections remain life-threatening to cystic fibrosis patients, and due to the limited eradication efficiency of current treatments, novel antimicrobial therapies are urgently needed. Surface proteins are among the best targets to develop new therapeutic strategies since they are exposed to the host’s immune system. A surface-shaving approach was performed using *Burkholderia cenocepacia* J2315 to quantitatively compare the relative abundance of surface-exposed proteins (SEPs) expressed by the bacterium when grown under aerobic and microaerophilic conditions. After trypsin incubation of live bacteria and identification of resulting peptides by liquid chromatography coupled with mass spectrometry, a total of 461 proteins with ≥2 unique peptides were identified. Bioinformatics analyses revealed a total of 53 proteins predicted as localized at the outer membrane (OM) or extracellularly (E). Additionally, 37 proteins were predicted as moonlight proteins with OM or E secondary localization. B-cell linear epitope bioinformatics analysis of the proteins predicted to be OM and E-localized revealed 71 SEP moieties with predicted immunogenic epitopes. The protegenicity higher scores of proteins BCAM2761, BCAS0104, BCAL0151, and BCAL0849 point out these proteins as the best antigens for vaccine development. Additionally, 10 of the OM proteins also presented a high probability of playing important roles in adhesion to host cells, making them potential targets for passive immunotherapeutic approaches. The immunoreactivity of three of the OM proteins identified was experimentally demonstrated using serum samples from cystic fibrosis patients, validating our strategy for identifying immunoreactive moieties from surface-exposed proteins of potential interest for future immunotherapies development.

## 1. Introduction

*Burkholderia cepacia* complex (Bcc) is a group of more than 26 related species, well known as opportunistic pathogens that can cause lethal respiratory tract infections among immunocompromised patients, particularly in cystic fibrosis (CF) patients [1]. In 2021, in Europe, the prevalence of Bcc chronic infections in CF adults (higher than 18 years) who have never had a transplant was around 2.4% [2]. However, in some European countries, Bcc belongs to the emerging respiratory pathogens with increasing prevalence, with the maximum prevalence observed at 13.8% in Serbia [2]. In Portugal, a higher prevalence of 7.4% was observed [2]. Additionally, Bcc bacteria are common contaminants of pharmaceutical and disinfectant products, causing several nosocomial outbreaks in immunocompromised individuals [3].

All species of the Bcc are able to cause infections, but the more frequently isolated from CF patients are *Burkholderia cenocepacia* and *Burkholderia multivorans* [4,5]. These infections often evolve into chronic infections characterized by a decline in pulmonary function and exacerbation periods. One of the major threats to CF patients, principally associated with *B. cenocepacia* infections, is the development of the cepacia syndrome, a rapid and often fatal necrotizing pneumonia [6]. These bacteria are known for their intrinsic resistance to most of the clinically available antibiotics [7]. This resistance, coupled with the patient-to-patient rapid spread of highly transmissible strains and the vast assortment of virulence factors, renders these chronic infections highly unpredictable, hazardous, and nearly untreatable [7,8]. Recently, the CFTR modulator TRIKAFTA^®^, has been approved to treat CF patients with one copy of the F508del mutation and revealed good results in improving patients’ lung function [9]. The sputum pathogen load, including Bcc infections, was lower in the first month of treatment and was followed by a 6-month steady state of lung function and bacterial load [9]. However, in some CF patients, *Pseudomonas aeruginosa* and Bcc infections were persistent after treatment, and it is still unclear if effective eradication after long-term treatment is possible.

To date, no effective strategy to eradicate Bcc bacteria from CF patients is available [10]. Strategies that allow the eradication or control of these devastating infections are required, and strategies non-reliant on antimicrobials, as in the case of immunotherapies, are gaining attention. Immunotherapies like active vaccination or passive immunization have several characteristics ideal for tackling of multidrug-resistant bacteria like Bcc, due to their specific activity and unlikelihood to lead to the development of new antimicrobial resistance [11]. The development of these new strategies for Bcc is still in its early stages, with a variety of antigens and approaches currently under study. In an endeavor to increase the number of potential antigens for these developments, our previous work focused on the optimization of a surfomics approach never used before in Bcc [12]. This approach involved the use of a protease to “shave” protein surface moieties from living bacterial cells, purification of these moieties, followed by liquid chromatography–tandem mass spectrometry (LC-MS/MS) analysis. This methodology overcomes the limitations associated with membrane protein poor solubility and gel-dependent methods [13]. The methodology allows the identification of surface-exposed proteins, an essential class of proteins for therapeutic strategies against bacterial infections, due to their ability to interact with the environment and exposure to the host immune system [14]. Additional information is also obtained, as the recovered peptides comprise the surface-exposed moieties of the identified proteins, the most accessible to the host immune system. The success of this approach impelled us to compare the surface-exposed proteins of the highly virulent and transmissible epidemic strain *B. cenocepacia* J2315 [15] when grown in conditions mimicking those found in the CF lung under aerobic or microaerophilic conditions. A low oxygen environment is an important environmental condition present in the CF lung, since during the advancement of the CF disease, the patient’s lungs become filled with a dense and sticky mucus layer, generating microaerophilic environments within the lung [16,17]. In fact, concentrations of oxygen present in infected CF lungs vary severely from atmospheric to almost zero, as demonstrated by the isolation of strict anaerobes at high densities in the sputum of these patients [18]. Furthermore, Bcc bacteria can thrive intracellularly in host cells, where the pathogen can face low pH inside phagolysosomes, deficiency of nutrients, and limiting levels of oxygen [19]. These unique conditions of reduced oxygen levels contribute to the decline of the lung function and to the persistence of infections, as the bacteria in this hypoxic environment have been found to differently express virulence factors and exhibit increased resistance to antimicrobials [16,20,21]. The significance of these environmental conditions in the context of the CF lung infection underscores the importance of detailed knowledge about surface-exposed proteins for the proper tailoring of immunotherapies. In the present work, a surface-shaving approach was used on *B. cenocepacia* J2315 cells grown under aerobic or microaerophilic conditions. The strain was chosen since it is the reference strain of the epidemic ET12 lineage, and its genome is publicly available [15]. This approach led to the identification of a total of 461 proteins. The proteins with predicted subcellular localization as outer membrane or extracellular were further analyzed, their predicted B-cell epitopes were compared to the surface-exposed moieties found, and their levels of expression under both aerobic and microaerophilic conditions were compared. Additionally, sera samples from CF patients with a clinical history of Bcc infection were tested for immunoreactivity against three selected OM proteins predicted to have surface-exposed moieties with predicted immunogenic B-cell linear epitopes and protegenicity higher than 90.

## 2. Materials and Methods

### 2.1. Bacterial Strains and Culture Conditions

The *B. cenocepacia* J2315 CF isolate was used in this work and maintained on PIA (Pseudomonas Isolation Agar, Becton Dickinson, Heidelberg, Germany) plates. The *Escherichia coli* LMG194 (Invitrogen, Carlsbad, CA, USA) was maintained in LB-Miller broth (containing in g/L, tryptone, 10; yeast extract, 5; NaCl, 10). The strains were cultivated at 37 °C in shaking flasks (250 rev/min) containing liquid LB-Miller with the appropriate antibiotics or on the surface of the previously described Artificial Sputum Medium (ASM) agar plates [22].

### 2.2. Surface Shaving of Live B. cenocepacia Cells and Peptide Extraction

The “shaving” and recovery of peptides from *B. cenocepacia* J2315 cells using trypsin were performed as described by Sousa et al. (2020) with slight adaptations [12]. Bacteria were inoculated with an initial optical density at 600 nm (OD600) of 0.1, and after four hours of growth, the OD600 was normalized to 0.5. 100 μL aliquots of the normalized bacterial suspensions were inoculated on the surface of ASM agar plates. The plates were incubated for 22 h at 37 °C under both aerobic and microaerophilic conditions. Microaerophilic conditions were attained using the CampyGenTM Compact Sachet in an OxoidTM Compact Plastic Pouch (Oxoid, Basingstoke, UK). To confirm the desired conditions, a plate containing Mueller Hinton agar (Sigma Aldrich) inoculated with *Campylobacter jejuni* ATCC 33560 was used as a control. After cultivation for 22 h, the cells were gently collected from the plate surface, washed thrice, and digested with trypsin, as previously described [12]. Then, the mixtures were centrifuged (3500× *g*, 5 min, 4 °C), and the supernatants were filtered using 0.22 μm-pore-sized syringe filters (Whatman^TM^, Buckinghamshire, UK). The filtrate is the surfome that was re-digested with 1 μg/mL trypsin overnight at 37 °C with gentle agitation and then stored at −80 °C until further processing. Three biological replicates of each total exoproteome were performed.

### 2.3. LC-MS/MS Analysis

The peptides were separated, as described previously [12], using a Dionex Ultimate 3000 nano UPLC (Thermo Scientific, San Jose, CA, USA), equipped with a reverse-phase C18 75 μm × 50 Acclaim Pepmap Column (Thermo Scientific), in 85 min chromatograms using a flow rate of 300 nL/min and 40 °C. Previously, the peptide mixture was concentrated and purified using a 300 μm × 5 mm Acclaim Pepmap cartridge (Thermo Scientific) in 2% ACN/0.05% formic acid for 5 min, at a flow of 5 µL/min. Chromatographic separation was accomplished using Solution A (0.1% formic acid) and Solution B (80% ACN, 0.1% formic acid) as the mobile phase, according to the following elution conditions: 4–35% solution B for 60 min; 35–55% solution B for 3 min; 55–90% solution B for 3 min. Then, an 8 min wash with 90% solution B, followed by re-equilibration for 12 min with 4% solution B, was carried out. Peptides were eluted and ionized by a nano-electrospray ionization source and were further evaluated using a trihybrid Thermo Orbitrap Fusion (Thermo Scientific) mass spectrometer working in positive mode, in Top30 Data Dependent Acquisition mode. Peptide precursors were attained in single MS mode, within a 400–1500 *m/z* range at 120,000 resolution (at 200 *m/z*) with a 4 × 10^5^ ion count target threshold. For MS/MS, precursor ions were formerly isolated in the quadrupole at 1.2 Da, and then CID-fragmentation occurred in the ion trap with 35% normalized collision energy. Monoisotopic precursor selection was turned on. Ion trap parameters were: (i) automatic gain control at 2 × 10^3^; (ii) 300 ms for maximum injection time; and (iii) sampling of only precursors with charge states 2–5 for MS/MS. A dynamic exclusion time was set to 15 s, with a 10 ppm tolerance around the selected precursor and its isotopes to avoid redundant fragmentations.

### 2.4. Protein Identification by Database Searching

Proteome Discoverer (version 2.5.0.400, Thermo Scientific) was used to process raw data from mass spectrometry without applying charge state deconvolution and deisotoping. The SEQUEST engine was used to examine MS/MS spectra against the *B. cenocepacia* J2315 genome database downloaded from Uniprot (www.uniprot.org; accessed at 22 March 2022). Search parameters applied were one missed cleavage after trypsin digestion; methionine oxidation as a variable modification; 10 ppm mass tolerance of precursor ions; and 0.1 Da of tolerance of product ions. Peptide identifications were accepted when higher than the filter parameter Xcorr score vs. charge state with SequestNode Probability Score (+1 = 1.5, +2 = 2.0, +3 = 2.25, +4 = 2.5). Validation of peptide spectral matches (PSM) was performed at a 1% FDR using percolator based on q-values.

### 2.5. Bioinformatic Analysis of Protein Sequences

Initial predictions of subcellular localization were achieved using PsortB v3.0 (https://www.psort.org/psortb/; accessed at 23 March 2023). When subcellular localization was unknown (U), a different analysis using LocTree3 was performed [23]. The presence of features like signal peptides was obtained using SignalP—6.0 (https://services.healthtech.dtu.dk/services/SignalP-6.0/; accessed at 23 March 2023). Proteins previously identified as cytoplasmic (C), cytoplasmic membrane (CM), or periplasmic (PM) were further analyzed for the probability of being moonlight proteins with secondary functions at the outer membrane or extracellularly. This analysis was performed using the Databases MoonProt 3.0 and MultitaskProtDBII. Proteins bioinformatically identified as extracellular (E) or outer membrane (OM) were selected for further analysis of B-cell linear epitopes using BepiPred-2.0, within the immune epitope database (IEDB), using a 0.5 threshold [24]. This threshold was chosen since it maximizes BepiPred-2.0 sensitivity and specificity. Only peptides ranging from 5 to 25 amino acids were considered, as this is the typical range of B-cell epitopes [24]. Vaxign2 (https://violinet.org/vaxign2; accessed at 27 April 2023) was used to predict the potential of the identified proteins as vaccine candidates using the principle of reverse vaccinology [25], and adhesion probability was calculated on the vaxign2 page using SPAAN software [26].

### 2.6. Production of Anti-Hfq2 and Anti-GroEL Polyclonal Antibodies

Nucleotides 350 to 538 of the *hfq2* (BCAL1538) gene and nucleotides 1192 to 1461 of the *groEL* (BCAL3146) gene were amplified from the *B. cenocepacia* J2315 genome with the primer pairs AMG1_Fw (GAATTCGCCGCGTGAAGGCTACGGTT) / AMG1_Rv (GTCGACTACTGGCCGTCCGGCACGAT) and AMG3_Fw (GAATTCGACGCACTGCACGCAACC) / AMG3_Rv (TTGTCGACGTACTCGCCCGTTGCT), respectively. The resulting amplicons, ranging in size from 228 to 284 bp, were digested with EcoRI and SalI and cloned into the same cloning sites of the pMAL-c2X vector (New England Biolabs, Ipswich, MA, USA), generating the plasmids pMAL-Hfq2 and pMAL-GroEL. These plasmids allowed the expression of the C-terminal Hfq2 or GroEL peptides, fused to Maltose-binding protein (MBP) at the N-terminus. The plasmids were sequenced to confirm correct insertion of the cloned products and transformed into *E. coli* LMG194. In order to produce polyclonal antibodies against Hfq and GroEL proteins, the constructions were sent to the commercial company SICGEN (Portugal), where the recombinant peptides were overexpressed, purified, and used to produce polyclonal goat antibodies. The antibodies were purified by immunoaffinity, and their specificity was tested by Western blot against total cell extracts of *B. cenocepacia* J2315.

### 2.7. Fractionation of Cell Proteins

*B. cenocepacia* J2315 cultures were grown as described for surface shaving. After 22 h, the cells were collected using PBS 1X. A volume of bacterial suspension corresponding to approximately an OD_600_ of 80 was harvested by centrifugation (7000× *g*, 4 °C for 30 min). The pellet was used for the fractionation of cytoplasmatic and outer membrane proteins, and the bacterial supernatant was used for the extraction of extracellular proteins, following a procedure derived from Wickramasekara et al. (2011) and Sandrini et al. (2014) [27,28].

For extracellular proteins, 12 mL of the bacterial supernatant was centrifuged under the same conditions and was mixed with ice-cold 10% (*w*/*v*) Trichloroacetic acid (TCA, Sigma) and incubated at 4 °C for 30 min. After centrifugation (14,000× *g* for 30 min), precipitated proteins were washed with cold acetone, the pellets were air-dried and dissolved in 75 μL of SDS sample buffer (100 mM Tris base pH 6.8, 4% (*w*/*v*) SDS, 20% (*v*/*v*) glycerol, 0.2% (*w*/*v*) bromophenol blue, 200 mM DTT), followed by heating for 5 min at 95 °C. For cytoplasmatic and outer membrane proteins, the pellet was washed twice with 10 mM Tris buffer (pH 7.5) and resuspended in 10 mL of this buffer. The sample was then frozen at −80 °C. The sample was thawed and lysed by ultrasonic vibration using a Branson sonifier 250 (Branson Ultrasonics, Brookfield, CT, USA), using 6 cycles of sonication of 30 s each at a 40% duty cycle. Cell debris and unlysed cells were removed by centrifugation (6700× *g*, 15 min, 4 °C), and the proteins of the supernatant were separated by ultracentrifugation (108,726× *g*, 15 min at 4 °C) using a Beckman XL-90 Ultracentrifuge. The pellet, containing total membrane proteins, was washed with 10 mM Tris buffer (pH 7.5). The supernatant, containing cytoplasmatic proteins, was also mixed with 0.2 volumes of SDS sample buffer and heated for 5 min at 95 °C. To separate the outer membrane proteins, the total membrane protein pellet was resuspended in 10 mM Tris buffer supplemented with 2% (*v*/*v*) Triton, and after 30 min of incubation, the mixture was ultracentrifuged (108,726× *g*, 15 min at 4 °C). The outer membrane pellet was washed with 10 mM Tris buffer (pH 7.5) and resuspended in SDS sample buffer.

### 2.8. Western Blot Analyses

Extracellular, cytoplasmatic, and outer membrane proteins were separated using 12.5% SDS-PAGE. A Trans-Blot^®^ SD (BIORAD, Hercules, CA, USA) device was used to transfer the proteins from the gel to NC membranes (PALL corporation). The membranes were then blocked overnight at 4 °C with 3% (*w*/*v*) Albumin (BSA) fraction V (PanReac AppliChem) in PBS 1×. The membrane was probed with the primary Goat antibody of interest, anti-HFQ2 (1:1500 dilution), anti-GroEL (1:3500 dilution), or anti-BCAL2645 [29] (1:4000 dilution) for 2 h at room temperature. Then, the secondary antibody HRP-conjugated Mouse anti-Goat IgG antibody diluted 1:10,000 (SANTA CRUZ biotechnology, Dallas, TX, USA) was added. After 1 h of incubation at room temperature, the membranes were treated with the peroxidase substrate ECL (Sigma, St. Louis, MO, USA). Chemiluminescence signals were detected using a FUSION Solo device (Vilber Lourmat, Collégien, France).

### 2.9. Cloning and Overexpression of B. cenocepacia J2315 bcal1985, bcal2645, and bcam1931

**Figure 1:**
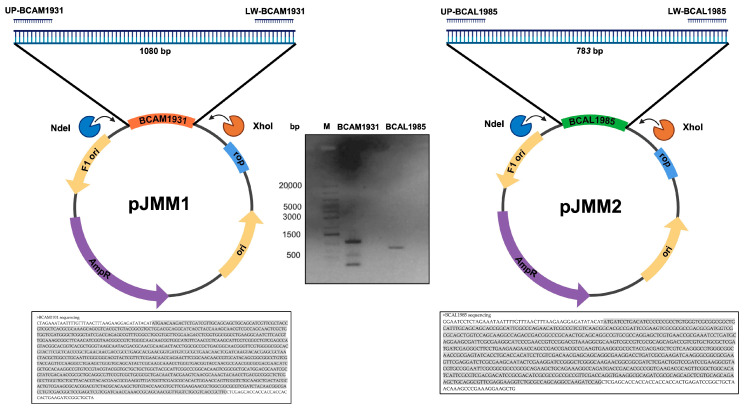
Schematic representation of the cloning of *bcal1985* and *bcam1931* genes in pET23a+, yielding the plasmids pJMM1 and pJMM2, respectively. In the middle, the agarose gel of the PCR products *bcam1931* and *bcam1985* is represented*. M—*Gene Ruler 1kb Plus DNA ladder (20,000–75 bp). The sequencing results from the pJMM1 and pJMM2 using the T7 promoter primer are represented in the bottom boxes.

The genes *bcam1931* and *bcam1985* PCR products were obtained using the primers pairs UP-BCAM1931 (5′-CGCCATATGAACAAGACTCTG -3′) and LW-BCAM1931 (5′-TATCTCGAGGAAGCGGTGACG-3′), and UP-BCAL1985 (5′-CGCCATATGATCCTGACATC-3′) and LW-BCAL1985 (5′-CTTCTCGAGCTGGATCTTGGC-3′), respectively. The NdeI and XhoI target sequences are underlined in each primer pair. The 1080 bp (*bcam1931*) and 783 bp (*bcal1985*) PCR products were digested with the restriction enzymes NdeI and XhoI, and the restriction fragments were ligated into the NdeI/XhoI-digested pET23a+, yielding pJMM01 and pJMM02, respectively. To confirm if the gene was present in the constructed plasmid without errors, plasmid DNA was extracted and sequenced using the T7 primer. With Blastn suite-2 sequences software, the nucleotide sequences of the genes *bcam1931* and *bcal1985* were fully aligned with the sequencing results, as shown in Figure 1. The constructed plasmids pJMM01 and pJMM02 allow for the controlled expression of C-terminus 6× His-tag derivatives of the proteins BCAM1931 and BCAL1985, respectively.

*E. coli* BL21 (DE3) transformed with pJMM01 or pJMM02 was cultured at 18 or 30 °C, respectively, in shake flasks, using SB supplemented with 150 µg/mL ampicillin. Induction with IPTG (0.4 mM), bacterial harvesting after 18 h (pJMM01) or 2 h (pJMM02) of cultivation, processing, and recombinant protein purification and western blot analysis were performed based on previously described methods [12].

### 2.10. Purification of His-Tagged Proteins BCAL1985, BCAL2645, and BCAM1931

Bacterial cell suspensions were disrupted by ultrasonic vibration using a Branson sonifier 250 (Branson Ultrasonics, Brookfield, CT, USA), with 5 cycles of 25 s each at 40% duty cycle. Before the last sonication cycle, 2% (*v*/*v*) Triton X-100 (BCAM1931) or Triton X-114 (BCAL1985) and 0.5 mM Phenylmethylsulfonyl fluoride (PMSF) were added to the bacterial suspensions for protein solubilization and inhibition of protein degradation by proteases, respectively. Non-soluble cell debris was removed by centrifugation for 30 min at 12,000× *g* and 4 °C. The cleared supernatants were collected and maintained at 4 °C until further processing. Both recombinant proteins were purified by affinity chromatography using HisTrap FF columns (GE Healthcare, Chicago, IL, USA). The columns were initially equilibrated with 10 mL of Buffer I supplemented with 1% (*v*/*v*) Triton X-100 (BCAM1931) or without detergent (BCAL1985) and 0.5 mM PMSF. Proteins were eluted with 5 mL of Buffer I containing increasing imidazole concentrations of 50, 60, 80, 100, and 200 mM (BCAM1931) or 60, 100, 200, 300, and 500 mM (BCAL1985). Aliquots of 1 mL were collected for each protein, followed by SDS-PAGE analysis. Western blot using a monoclonal anti-polyhistidine peroxidase conjugate clone HIS-1 antibody (diluted 1:2000, Sigma, St. Louis, MO, USA) was performed, as previously described [12]. BCAL2645 purification was carried out, as previously described [12].

### 2.11. CF Patients’ Blood Sera Immunoreactivity Assay against BCAL1985, BCAL2645, and BCAM1931 Proteins

The purified 6 × His-tagged BCAL1985, 6 × His-tagged BCAL2645, 6 × His-tagged BCAM1931, and BSA were loaded into 12.5% SDS-PAGE gels and electrophoresed at 160 V. Western blot and chemiluminescence signal detection were performed, as described previously [12]. Serum samples SCF1 and SCF2 were gathered from two CF patients with positive cultures of Bcc bacteria who received treatment at Hospital Santa Maria (Lisbon, Portugal). Serum samples SCF3 and SCF4 were acquired from two CF patients with positive cultures of Bcc at Hospital de D. Estefania (Lisbon, Portugal). The serum samples were obtained from the blood samples collected, as previously described [12].

### 2.12. Data and Statistical Analysis

Peptide extractions from both the microaerophilic and aerobic conditions were performed in triplicate from three independent cultures and “shaving” experiments. Proteins considered had to be identified in at least two of the samples, with proteins found in only a single sample being discarded from the overall count of identified proteins. Excel (Microsoft Excel 2021 v16.72 for Mac, Microsoft, Redmond, WA, USA) was used for means and standard deviations calculations to perform quantitative analysis. Values were z-scored prior to heatmap analysis. Heatmaps were made with the ClustVis web tool (https://biit.cs.ut.ee/clustvis/; accessed at 7 November 2023) using default parameters. A 0 value was assigned to non-detected proteins in samples to avoid processing unavailable data. PCA analysis was performed with Proteome Discoverer (version 2.5.0.400, Thermo Scientific).

## 3. Results and Discussion

### 3.1. “Shaving” of Surface-Exposed Proteins of B. cenocepacia J2315 Live Cells

**Figure 2:**
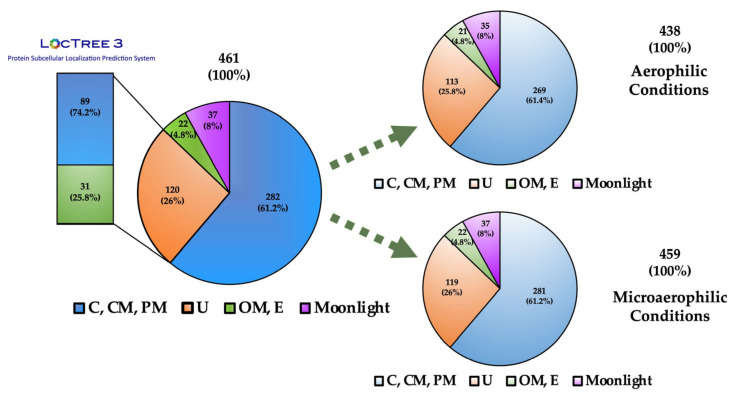
Schematic representation of the subcellular localization of the proteins identified using the surface-shaving approach, with the number of proteins identified in each environmental condition represented. The programs PsortB v3.0 and LocTree3 were used to predict the subcellular localization. Moonlight proteins with secondary functions at the outer membrane or extracellularly were identified using the Databases MoonProt 3.0 and MultitaskProtDBII. Localization: (C) cytoplasm; (CM) cytoplasmic membrane, (PM) periplasm; (U) unknown; (OM) outer membrane; (E) extracellular.

*B. cenocepacia* J2315 live cells were cultured on ASM under either microaerophilic or aerobic conditions, with the first condition being more closely related to the environment found in the CF lung at later stages of the disease. The cells were submitted to trypsin digestion using previously optimized conditions that minimize cell lysis and contamination with cytoplasmic proteins [12]. This short digestion time may result in large protein fragments that are not identifiable by MS/MS. Therefore, a re-digestion step was performed in which the supernatant containing the proteins’ digested fragments was digested for a longer period of time (overnight). The resulting peptides (surfome) were recovered and analyzed by LC-MS/MS. A total of 461 proteins with ≥2 unique peptides that were identified in at least two independent duplicates were found in the collective samples under aerobic and microaerophilic conditions, with 459 found under microaerophilic conditions and 438 under aerobic conditions (Figure 2, Tables S1–S5).

A total of 53 proteins were predicted to be surface-exposed based on their outer membrane (OM) or extracellular (E) localization after the analysis of subcellular localization using PsortB v3.0 combined with LocTree3 prediction programs (Figure 2; Table 1 and Table 2; Tables S2 and S4). In the surfome, a total of 408 proteins were found to be in silico predicted to be not exposed on the bacterial surface. The analysis of subcellular localization using PsortB v3.0 combined with LocTree3 programs predicted 267 proteins to be only localized in the cytoplasm (C), 24 proteins on the cytoplasmic membrane (CM), 116 proteins in the periplasm (PM), and 1 protein with unknown (U) localization (Tables S3 and S4). The genome of *B. cenocepacia* J2315 has a total of 7117 coding sequence (CDS), with 3138 predicted to be localized in the C fraction, 1518 in the CM fraction, 235 in the PM fraction, and 289 localized on the bacterial surface or extracellularly [30]. Considering the total number of CDS for each subcellular fraction, the surfome samples have 8.5% of the C fraction, 1.6% of the CM fraction, 49.4% of the PM fraction, and 18.3% of the bacterial surface or extracellular fraction. These results shown minor contamination with the C and CM fractions; however, a high number of PM proteins appear in the surfome samples extracted. Nevertheless, these values do not take into consideration the protein expression profile under each tested environmental condition.

**Figure 3:**
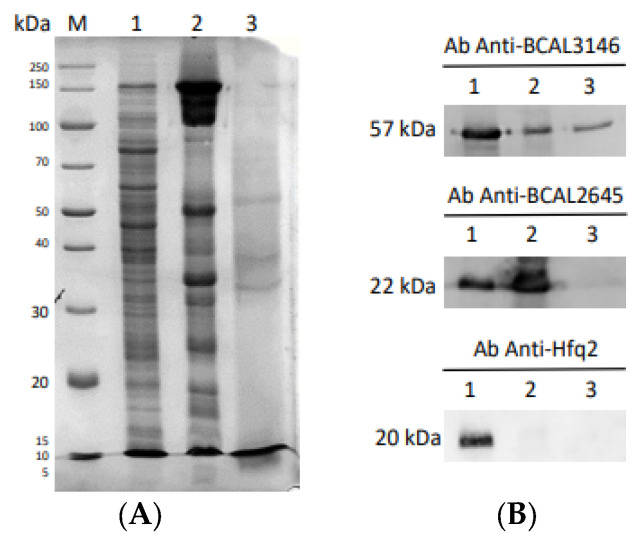
Subcellular localization of *B. cenocepacia* J2315 BCAL3146 (GroEL), BCAL2645 (OmpA), and BCAL1538 (HFQ2). After bacterial growth in ASM medium under aerobic conditions, cells were harvested and divided into the following fractions: Cytoplasmic proteins (Lane 1), Outer Membrane proteins (Lane 2), and Extracellular proteins (Lane 3). These fractions were analyzed by SDS-PAGE (**A**) and by Western blot using the goat IgG antibodies anti-BCAL3146, anti-BCAL2645, or anti-Hfq2 (**B**). M-PageRuler^TM^ unstained broad-range protein ladder (Thermo Scientific).

To validate the subcellular localization of the proteins identified in the surfome obtained through the surface-shaving approach, the subcellular fractionation technique was employed to determine the localization of GroEL, BCAL2645. and HFQ2 proteins. The GroEL and HFQ2 proteins were predicted in silico to be localized in the C fraction, while the OmpA-like protein BCAL2645 was predicted to be localized on the OM. The GroEL and BCAL2645 peptides were identified in the surfome analysis by LC-MS/MS under all tested environmental conditions, while the HFQ2 protein was not detected (Table S1). To confirm these results, the *B. cenocepacia* J2315 cells were grown in ASM medium under aerobic conditions and fractionated into the subcellular fractions C, OM, and E. The obtained proteins were size-separated by SDS-PAGE, electroblotted onto nitrocellulose, and detected using a goat IgG antibody specific for each protein (Figure 3). The BCAL2645 protein was found in the C and OM, as expected for an OmpA-like protein and as described in other bacteria [31]. The RNA chaperone HFQ2 was exclusively detected in the C fraction [32]. This result was anticipated, since the Hfq2 protein was not detected in the extracted surfome samples. However, in contrast with the in silico prediction, the molecular chaperone GroEL was found in all protein subcellular fractions tested. In fact, this cytoplasmic protein was found in the surfome samples under both conditions tested. The intracellular chaperone GroEL, associated with protein folding, has been found in several bacteria on the cell surface or secreted during host cellular invasion, being recognized by the host immune system as an antigen [33]. In the *Burkholderia* genus, GroEL has been previously found in the extracytoplasmatic fraction and was able to elicit the host immune system [34,35,36].

The results obtained for the GroEL protein suggest that other surfome-identified proteins predicted in silico to be in the C, cytoplasmatic membrane (CM) fraction, periplasm (PM), or unknow (U) subcellular fraction can have moonlight functions on the bacterial surface. Therefore, the 408 proteins were analyzed using the Databases MoonProt 3.0 and MultitaskProtDBII. A total of 37 proteins, comprising 29 from the C and 8 from the PM fraction, were identified as putatively possessing moonlight functions on the bacterial surface of other bacterial species and with roles in the adhesion or invasion of host cells (Table S5). The majority of the moonlight proteins found have canonical function as metabolic enzymes (29 proteins). Six Enzyme Commission (EC) groups are represented among the proteins identified as possessing putative moonlight activity, with 8 enzymes from group 1 (oxidoreductases), 7 from group 2 (transferases), 4 from group 3 (hydrolases), 2 from group 4 (lyases), 7 from group 5 (isomerases), and 1 from group 6 (ligases). One group of these enzymes is involved in cell redox homeostasis (peroxiredoxins BCAL1070, BCAL3192, and BCAL2013, and SodB). Other moonlight proteins identified are molecular chaperones/heat shock proteins (GroEL, DnaK, ClpA, ClpB, GrpE), involved in protein synthesis (TufA, FusA) or in the bacterial secretion system (SecA). Non-moonlight periplasmic and cytoplasmic proteins most probably arose in surfomic samples as a result of cell lysis during the incubation of living cells with trypsin.

### 3.2. Surfome Proteins Predicted to be Located at the Outer Membrane

Of the 53 proteins identified to have subcellular localization at the bacterial surface, 21 were predicted to be localized in the OM or to belong to the fimbriae or flagella structures (Table 1).

Vaxign-ML was used to estimate the protegenicity (protective antigenicity) scores of the identified proteins. The majority of proteins included in approved vaccines already licensed or in clinical trials have a protegenicity score higher than 90 [25]. Using this criterion, we predict that all the proteins identified, except for BCAS0522, are good antigen candidates for vaccine development. The proteins BCAM2761 and BCAS0104 presented the highest scores (>95). BCAM2761 encodes the pilus major subunit CblA in ET12 *B. cenocepacia* strains, which is associated with a 22 kDa adhesin, shown to be involved in the persistence of infection and the development of severe inflammation in CF patients [37]. BCAS0104 encodes the structural component of the flagella, the cap protein FliD2, which is a paralog of BCAL0113 [30]. In addition, flagellar cap proteins can bind to mucins, which is important for the colonization step of the CF lung.

When considering the predicted B-cell epitopes, 15 of these OM proteins were shown to have at least one B-cell epitope with more than 5 consecutive amino acids in the surface-exposed moieties of the protein, reinforcing their probability of eliciting a B-cell immune response during infection. Most of the proteins identified have exposed B-cell epitopes in both growth conditions tested (10 proteins). However, 3 of them have only B-cell epitopes under aerobic conditions (BCAL0304, BCAL0349, BCAM2549) and 2 of them under microaerophilic conditions (BCAL0565, BCAS0104). The proteins BCAL0577, BCAL1893, BCAL2820, BCAM2418, BCAS0236, and BCAS0522 have no exposed B-cell epitopes predicted.

Flagellar hook-associated protein FlgL (BCAL0577) homologs have been used as carrier proteins in gold nanoparticle-vectored LPS glycoconjugates vaccines against melioidosis and glanders [38]. However, their protective efficacy was dubious. BCAM2418 homolog protein BimA was also used as an antigen in a vaccine study using BCALB/c mice and *B. mallei *or*B. pseudomallei*challenge[39]. In these studies, it was shown that all vaccinated animals survived 21 days post-challenge compared with only 12.5% in the control animals. Later, using BimA with cationic liposome-DNA complex and*B. pseudomallei*challenge, these authors revealed ~80% protection in the acute phase of infection and ~20% long-term protection. TheBCAL3204 homolog Pal (BPSL2765) was previously shown to be involved in*B. mallei’s*ability to resist complement-mediated killing and to replicate inside host cellsin vitro*.*The protein was also able to induce host antibodies during the course of infection and contributed to the bacterium’s virulence in a mouse model of infection[40]. In this study, the authors also showed that Pal protein administration to mice led to an 80% survival over a period of 40 days post-challenge with*B. mallei.*

To achieve successful colonization, bacterial pathogens encode adhesins to mediate their adherence to host cell surface receptors and extracellular matrix components [41]. Using the minimum threshold of 0.51, 17 proteins were identified with possible roles in adhesion to host cells. To identify top-scoring novel adhesins with high confidence using the SPAAN program, we used a stringent criterion of *P*_ad_ > 0.7 to reduce the detection of false positives. Using this criterion, only 10 proteins were identified, being associated with flagella (BCAL0565, BCAL0577, BCAS0104), pilus (BCAM2761), type VI secretion systems (BCAL0349), protein transport (BCAL3203), transmembrane transport (BCAM1931), OM integrity (BCAL3426), or trimeric autotransporter adhesins (BCAM2418, BCAS0236). High levels of mRNA corresponding to *BCAM2418* and *BCAS0236* were found in cells of *B. cenocepacia* K56-2 after physical contact with bronchial epithelial cells [42]. In addition, an anti-BCAM2418 antibody was shown to inhibit cellular adhesion of *B. cenocepacia* strains to bronchial cells and mucins and to exert an inhibitory effect when the bacterium infected *Galleria mellonella* [43]. The anti-BCAM2418 antibody used specifically targets the passenger domain of BCAM2418 (aa 100–234), which is extracellular and normally involved in virulence. In fact, in the surfome samples analyzed, only the amino acid residues 205–282 of the total 2775 amino acids of the BCAM2418 protein were identified (Table S6). As expected, these results indicate that under conditions mimicking the CF lung, the BCAM2418 passenger domain was extracellularly located.

Blocking the initial stages of infection, namely, bacterial attachment to host cells or/and the mucosal surface, can be an effective strategy to avoid bacterial infections. Further studies on these 10 identified proteins could pave the way to the development of new immunotherapeutic strategies targeting the proteins involved in the initial stages of infection and abolishing the colonization process [44].

### 3.3. Surfome Proteins Predicted as Extracellular

Of the 53 proteins identified to have subcellular localization at the bacterial surface, 32 were predicted to be E (Table 2).

Using a protegenicity threshold of 90, 20 proteins were predicted as good candidates for vaccine development, with the proteins BCAL0151 and BCAL0849 having the highest scores (>95). BCAL0151 is an extracellular ligand-binding protein, and BCAL0849 is an M48B metallopeptidase. BCAL0849 was previously demonstrated to be upregulated in *B. cenocepacia* J2315 grown in CF sputum samples, with possible roles in host tissue destruction [45].

When considering predicted B-cell epitopes, 24 of these proteins were shown to have at least 5 consecutive amino acids coinciding between their B-cell epitopes and the peptides found through the surface-shaving approach, reinforcing their probability of eliciting a B-cell immune response during infection. Most of the proteins identified have predicted exposed B-cell epitopes under both growth conditions tested (19 proteins). However, epitopes could only be predicted under microaerophilic conditions for 5 of them (BCAL0360, BCAL0562, BCAL0849, BCAL1390, BCAL1938). Eight of these proteins have no predicted exposed B-cell epitopes; 6 of them are classified as hypothetical proteins.

BCAL0343 homolog protein Hcp6 has been studied as a potential melioidosis vaccine antigen [46].It was shown that recombinant Hcp6 protected 50% of mice against *B. pseudomallei* challenge.

### 3.4. Quantitative Analysis of Proteins Predicted as Outer Membrane or Extracellular Proteins

**Figure 4:**
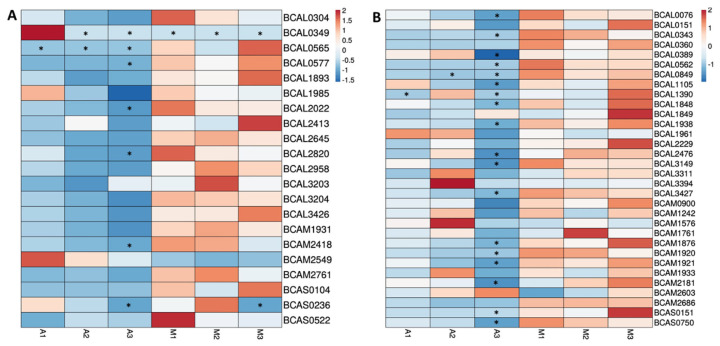
Heatmaps of z-scored abundances of identified OM, fimbrial, and flagellar proteins (**A**), and extracellular proteins (**B**) in the surface-shaving approach. Proteins are shown in rows, and the surfomics samples are shown in columns in each heatmap. A1, A2, A3 refer to aerobic environment, and M1, M2, M3 refer to microaerophilic environment. The numbers next to the growth condition tested represent each of the three biological replicates. Increasing intensity in the positive range (Orange) exemplifies abundances that are greater than the mean abundance resulting from both conditions relative to the standard deviation associated with the mean. Increasing intensity in the negative range (Blue) represents abundances that are lower than the mean abundance derived from both conditions relative to the standard deviation associated with the mean. * Surfomic samples with no peptide identified for the protein in the analysis.

The relative abundance of the 53 proteins with predicted function or localization at the bacterial surface or extracellularly was analyzed (Figure 4). Nine out of the 53 proteins are highly abundant (>30 PSMs), with four of them belonging to the OM group (BCAL2958, BCAM1931, BCAM2761, and BCAS0104) and five with predicted E localization (BCAL0151, BCAL1961, BCAL2229, BCAL3311, and BCAM0900).

Most of the proteins had more PSMs in surfomic samples from cells grown under microaerophilic conditions, with the most abundant proteins being BCAL0343, BCAL0360, BCAL0562, BCAL0577, BCAL0849, BCAL2645, BCAM1920, BCAM2761, BCAS0104, and BCAS0151. BCAL0565 was only present in surfomic samples under microaerophilic conditions, while BCAL0349 was only present under aerobic conditions. BCAL0349 was present in samples A1 and A2; however, it was only possible to quantify sample A1. BCAL0849 and BCAL1390 could only be quantified in one sample from aerobic surfomic replicates; therefore, the presence of these proteins in cells grown under aerobic conditions is not clear.

In the work of Sass et al. (2013) [19], the transcriptomic profiling of *B. cenocepacia* J2315 revealed the upregulation of *BCAL0577*, *BCAL2645*, and *BCAL0562* under low oxygen concentration growth conditions. These authors also reported that *BCAL034*9 was downregulated under low oxygen concentration growth conditions. These results reinforce that higher surface-exposed moieties of these proteins are available in the surfomic samples under microaerophilic or aerobic growth conditions due to higher expression of these proteins. However, the majority of the proteins with higher PSMs were not detected as overexpressed under microaerophilic conditions by Sass et al. (2013) [19]. Therefore, the higher number of PSMs can be also due to a higher exposure of the protein moieties to trypsin under microaerophilic conditions.

### 3.5. Characterization of B. cenocepacia BCAL1985, BCAL2645, and BCAM1931 Proteins’ Immunoreactivity

To validate the findings obtained through our surfomics approach, we randomly selected three OMP proteins with a protegenicity score higher than 90 and with surface-exposed protein moieties containing predicted B-cell linear epitopes. The selected proteins were BCAL1985, a putative exported isomerase, BCAM1931, a putative porin, and BCAL2645, an OmpA-like protein. These details can be found in Table 3. The imunoreactivity of BCAL1985 and BCAM1931 was tested using sera samples from CF patients with a clinical history of Bcc infection, using BCAL2645 as a positive control, which was previously immunoreactive against sera samples SCF2 and SCF3 [12]. BSA was used as a negative control.

**Figure 5:**
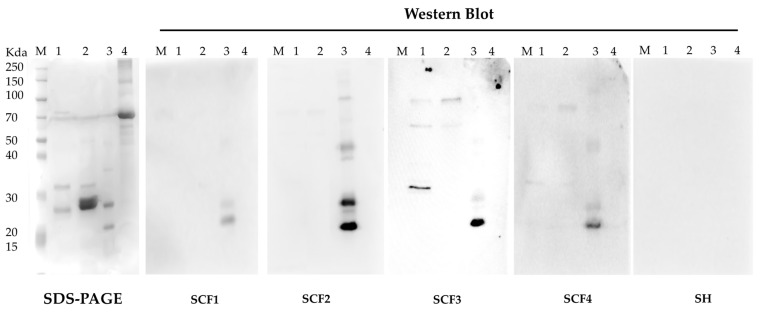
SDS-PAGE and Western blot of purified recombinant proteins BCAM1931, BCAL1985, and BCAL2645 from *B. cenocepacia* J2315 probed with the human serum samples SCF1, SCF2, SCF3, SCF4 obtained from CF patients infected with Bcc bacteria or a pool of human serum sample from healthy donors, SH. Lanes: M—PageRuler Unstained Broad Range Protein Ladder (Thermofischer); 1—purified BCAM1931 recombinant protein; 2—purified BCAL1985 recombinant protein; 3—purified BCAL2645 recombinant protein; 4—BSA.

The purified fraction of each protein was employed to identify IgG antibodies’ reactivity against each protein in 4 serum samples from CF patients with active Bcc infection (Figure 5). BCAL2465 protein exhibited strong immunoreactivity as it reacted with all tested sera, indicating that the protein is highly exposed to the host in different infections settings. On the other hand, the BCAM1931 protein showed high immunoreactivity against SCF3, lower immunoreactivity against SCF4, and no reactivity against the other two sera. As this gene is highly conserved in Bcc, these results suggest that although this protein is immunoreactive, its expression and surface exposure are not common traits during Bcc bacterial infection of CF patients. Unfortunately, no information regarding the strain causing the infection or the general wellbeing of the patients is known for any of the sera; as such, no correlation can be suggested. Previously, the BCAM1931 homolog OpcP was tested asa gold nanoparticle-coupled LPS glycoconjugate carrier protein[47]. OpcP was able to elicit significant levels of protection with a 77% survival rate. However, the surviving animals exhibited colonization of the lungs, liver, and spleen, suggesting that dissemination was not prevented.

Lastly, the BCAL1985 protein was also tested, but no immunoreactivity was detected against any of the tested sera. This suggests that despite the presence of this protein on the surface of the bacteria, during the infection process, it might not be recognized by the host immune system due to differential expression or surface exposure in the infection setting. Another possible reason for the observed results may be related to the incorrect folding of the protein during its expression in *E. coli*, thereby hiding possible epitopes. Using samples of a pool of sera from healthy individuals, no reactivity against the tested proteins was observed (Figure 5).

These results demonstrate that this method proved to be effective, enabling the identification of putative immunogenic proteins. It should be noted that even though the immunoreactivity of only three proteins was tested in this work, some of the identified proteins have already been proven to be exposed to the host in an infection setting, as is the case of BCAL2958, BCAL2022, BCAL3204, BCAL3146, BCAL1893, and BCAL2820 [12,35,36]. While this method enables the identification of new immunogenic bacterial surface-exposed proteins, it is important to note that not all identified proteins are necessarily always expressed or exposed to the host during infection, as demonstrated by the lack of immunoreactivity of BCAL1985.

## 4. Conclusions

Bcc chronic infections remain a life-threatening concern for CF patients, with no current available treatment. The discovery of new strategies for the prevention of Bcc chronic infections is vital. Bacterial surface-exposed proteins have been studied as potential antigens for antibody-based therapy development. So, this study was centered on the identification of immunogenic surface-exposed proteins, with the aim of finding good candidates for active and passive immunotherapies. With this aim, a surface-shaving experimental technique using trypsin combined with protein moieties analysis by LC-MS/MS was conducted under both aerobic and microaerophilic growth conditions to gain further insights into possible immunotherapy candidates. A total of 90 proteins with predicted function or localization at the bacterial surface or extracellularly were identified using this approach. In silico analysis of these proteins revealed several proteins with high protegenicity scores and other proteins with a high probability of having important roles in bacterial attachment to host cells or/and the mucosal surface. A total of 71 surface-exposed B-cell linear epitopes were also identified.

However, the present work was performed using only 1 strain of *B. cenocepacia*, so future work using a similar methodological approach with several clinical CF strains will be helpful to validate the selection of the best bacterial protein targets for the future development of active and/or passive immunization strategies. A larger screen with a higher number of serum samples from CF patients with a clinical record of Bcc infection against some of the identified surface-shaved proteins will be also helpful in validating the target bacterial surface exposition under the host immune response. Furthermore, validation of the present target proteins’ bacterial surface exposition under other important CF lung environment conditions, such as in the presence of other common CF pathogens or after prolonged CF lung colonization, will be important.

In summary, this work allowed for a deeper understanding of surface protein expression in Bcc strains under conditions mimicking some of the CF lung environment conditions, and the surface-exposed moieties of these proteins were identified. This information will be valuable for the future development of highly specific antibodies capable of targeting Bcc colonization steps and the establishment of chronic infection or for the development of active immunization strategies for the prevention of Bcc infection. It is also worth mentioning that the identification of immunogenic proteins does not warrant the success of a vaccine or therapeutic antibodies. Further studies are required to exploit this information and develop such protective therapies.

## Figures and Tables

**Table 1:** Summary of *B. cenocepacia* J2315 proteins identified with the surface-shaving strategy followed by LC-MS/MS (threshold score ≥ 2 peptides), with a subcellular localization of OM, fimbrial, or flagellar. Data show the number of unique peptides identified, Vaxign-ML score, adhesin probability, and identified surface-exposed protein moieties containing predicted immunogenic epitopes, which are shown in bold. HP—Hypothetical protein.

ORF ^1^	Product ^1^	PSMs ^2^	Unique Peptides ^2^	Vaxign-ML Score ^3^	Adhesin Probability (Pad) ^4^	Surface-Exposed Protein Moieties with Predicted B-Cell Linear Epitopes ^5^
BCAL0304	VacJ-like lipoprotein	9	2	90.9	0.513	^195^**ANLLGAGDV**LDAAALDK^211 ○^
BCAL0349 *	Type VI secretion system-associated protein TagL	5	2	94.8	0.756	^195^**VSASEQGVL**DQTLANR^210 ○^ ^257^**TSNIAL**SQAR^266 ○^
BCAL0565	Flagellar basal body rod protein (FlgC)	6	3	90.9	0.852	^47^QVVFATD**PMGGARTASGQGVGGV**R^70 ●^
BCAL0577	Flagellar hook-associated protein (FlgL)	18	4	94.3	0.846	-
BCAL1893	Family M23 peptidase	7	3	91.3	0.645	-
BCAL1985	Putative exported isomerase	26	6	90.9	0.517	^77^E**GIPNRPD**VK^86 ◑^ ^235^AQIAQ**QLVQQKLQAFEEG**LR^254 ◑^
BCAL2022	PspA/IM30 family protein	19	5	90.9	0.406	^13^GLLNDAA**DSVQDPS R**^27 ◑^ ^150^DVAASA**LGGIGGKNLSEDFQK**^170 ◑^
BCAL2413	HP	17	3	90.9	0.639	^70^QMLFVDTVS**ASGAR**^83 ◑^ ^102^**DEIADPK**^108 ◑^
BCAL2645	Putative OmpA family membrane protein	22	3	90.9	0.623	^79^LAPSAAQTG**TQVTEQ**PDGSLK^99 ●^ ^178^LSAQG**MGASNPIADNATEAGR**^198 ◑^
BCAL2820	RND-4 efflux system outer membrane protein (oprM)	6	3	94.9	0.537	-
BCAL2958	Putative ompA family protein	78	9	92.8	0.581	^93^ITYQADAL**FDFDKATLKPLGKQ**KLDELASK^122 ◑^ ^140^**IGSDKYNDR**^148 ◑^ ^203^RVEVEVVG**TQQVQK**^216 ◑^
BCAL3203 ^#^	Tol-Pal system protein (TolB)	16	4	93.7	0.832	^176^YQLQISDSD**GQNAR**^189 ◑^
BCAL3204	Putative OmpA family lipoprotein	25	4	90.9	0.391	^97^HVLIQGNT**DERGTSEYNLAL**GQK^119 ◑^
BCAL3426	Putative lipoprotein (SlyB)	12	3	90.6	0.798	^56^**IQSDGGGSAIGTL**GGGALGAVA**GSAIGGGK**^85 ○^ ^128^SITQAAS**GEAFR**^139 ◑^
BCAM1931	Putative porin	62	10	92.5	0.915	^44^**SLWSMGSGID**QSR^56 ◑^ ^61^GS**EDLGGG**LK^70 ◑^ ^199^LGAAYS**QANLGDGTNANGATNIAAQGR**^225 ◑^
BCAM2418 ^#^	Trimeric autotransporter adhesin	26	3	93.05	0.898	-
BCAM2549	Multidrug efflux system outer membrane protein (OpcM)	17	6	92.5	0.305	^185^**ADQAQSEAL**FR^195 ○^ ^253^AKNELASAQAD**AVGVAR**^269 ○^
BCAM2761	Giant cable pilus (cblA)	49	6	95.9	0.929	^80^LATAP**ALKNQTSPGAA**EIPLSVK^102 ◑^
BCAS0104	A-type flagellar hook-associated protein 2 (HAP2) (fliD2)	30	9	96.6	0.884	^52^VATLA**ASQASGNT**R^65 ●^ ^481^MN**TNSQYLTR**LFG**GANSNGTL**SK^503 ●^
BCAS0236 ^#^	Trimeric autotransporter adhesin	6	2	92.2	0.917	-
BCAS0522	HP	14	2	82.2	0.471	-

^1^ *Burkholderia* Genome Database. ^2^ Data retrieved from LC-MS/MS analysis using Proteome Discoverer Program. PSM—Peptide Spectrum Matches. ^3^ Vaxign-ML score is the percentile rank score from the final Machine Learning classification model—recommended threshold: 90.0. ^4^ Calculated using SPAAN program with default settings—recommended threshold: 0.51. ^5^ Peptides identified by LC-MS/MS were analyzed for B-cell linear epitopes using http://tools.iedb.org/bcell/ (accessed at 29 May 2023). Threshold of 0.5. B-cell epitopes shorter than 5 or larger than 25 amino acids were not considered. * Subcellular classification of outer membrane using LocTree3. ^#^ Protein with multiple localization sites. ^○^ Only found under aerobic conditions. ^●^ Only found under microaerophilic conditions. ^◑^ Found under both conditions.

**Table 2:** Summary of predicted extracellular *B. cenocepacia* J2315 proteins identified using the surface-shaving strategy and LC-MS/MS (threshold score ≥ 2 peptides). The numbers of unique peptides identified, the Vaxign-ML score, and identified protein moieties containing predicted immunogenic epitopes (in bold) are shown. HP—Hypothetical protein.

ORF ^1^	Product ^1^	PSMs ^2^	Unique Peptides ^2^	Vaxign-ML Score ^3^	Surface-Exposed Protein Moieties with Predicted B Cell Linear Epitopes ^4^
BCAL0076 *	Putative lipoprotein	7	2	48.2	^86^**QEANDMSAQHNGGLSGDEQR**^105^ ^◑^
BCAL0151 ^#^	Extracellular ligand binding protein	90	15	98.9	^73^ITLQLDPQD**DAADPRQATQV**AQK^95 ◑^ ^119^IYSDAGVVQISPSATNP**AYTQQG**FK^143 ◑^ ^199^VMS**HDATNDKAVD**FR^213 ○^ ^325^A**NSTDPAK**ILAAMPATKYTGVIGTTTFD**S**^353 ◑^
BCAL0343	Putative type VI secretion system protein (TssD)	9	4	90.9	^30^S**WDHSIVQPR**^39 ●^ ^40^**SATASTAGGHTMTR**^53 ◑^
BCAL0360 *	HP	12	3	81.7	^48^D**NAPLDER**^55 ●^ ^72^AANHQVIG**TSETYSSVQA**R^90 ●^
BCAL0389 ^#^	Thiol:disulfide interchange protein (DsbC)	5	2	91.9	^40^**LGNDAPIK**^47 ◑^ ^224^**RLPGAVSADQL**NQALASSK^242 ○^
BCAL0562	Flagellin synthesis anti-sigma-28 factor (FglM)	8	4	90.9	^22^APSGTAQSSAQAGDAGSTGGDTTVNLSGL**SGQLR**^55 ●^
BCAL0849	Metallo peptidase, subfamily M48B	13	2	95.9	^159^SAAGAASPGVAAL**SSSQLGDIT**EK^182 ●^
BCAL1105 *	HP	10	2	90.4	^37^**DAMGH**DAMAK^46 ◑^
BCAL1390 *	Glucanase	3	2	90.9	^267^**ADPLA**APLLAK^277 ●^ ^365^FGADG**TLDTR**^374 ●^
BCAL1848 *	HP	6	2	87.0	^98^**AP**QALVVTT**RSAGSGGYVG**AQAYVTTSR^125 ◑^
BCAL1849 *	HP	13	4	90.9	^72^**GGGTGQ**LEYTVK^83 ◑^ ^153^**GVA**NVQLSFQA**AAPK**^167 ◑^
BCAL1938 *	Cysteine peptidase, family C40	3	2	90.9	^346^**TSTADDPIAR**^355^ ^●^
BCAL1961 *	HP	51	11	91.4	^58^**LDPNTLAPNG**DPILVIAAR^76 ◑^ ^82^VAAAIATTPN**VDLEK**^96 ○^ ^82^VAAAIATTPNVDLEKEDK^99 ◑^ ^174^GNHASTVTLLLDQ**GADPQ** **VK**^193 ○^ ^194^**NQLGIT**ALEFAK^205 ◑^ ^223^**IGASTPADAQK**^233 ◑^
BCAL2229 *	HP	36	11	90.9	^292^VGIIDLAS**RKLVQ**TIAVGR^310 ◑^
BCAL2476 *	HP	11	4	80.8	^72^**KFIIDD**NLK^80 ◑^
BCAL3149 ^#^	HP	10	4	77.0	^122^NV**HALQQGGATVTEGEEAVGG**R^143 ◑^
BCAL3311	BcnA	35	8	94.4	^64^AAQGSAQMTIDVAS**FDLGDKMYNDQVAGK**^92 ◑^ ^157^S**AFNVGTGEWKDTSIVA**DEVQIK^179 ◑^
BCAL3394 *	Putative exported ribonuclease	9	4	84.7	-
BCAL3427 *	Histone H1-like protein (HctB)	16	2	90.8	-
BCAM0900 *	HP	31	8	82.8	-
BCAM1242 *	HP	12	5	90.9	-
BCAM1576	Phosphoesterase family protein	29	14	93.7	^145^IT**DAQGKPL**PNGVITR^160 ○^ ^256^**VEGDDPAG**TR^265 ◑^ ^268^LADDS**PASALDGPPK**^282 ◑^
BCAM1761 *	Putative lipoprotein	14	2	84.9	^52^L**SGTEQSQHNGVTD**IAVGSNSYFVTLTPSGNGSVIK^87 ○^ ^91^**GSGSEPAEEAMR**^102 ◑^
BCAM1876 *	HP	15	4	89.7	-
BCAM1920 *	HP	7	3	90.9	^13^**ATLSSDSGSIR**^23^ ^◑^
BCAM1921 *	Putative phage membrane protein	13	2	90.9	^330^**LGGQVSNDVV**YAR^342 ◑^
BCAM1933 *	Putative cyclase	28	5	88.7	^33^**DLAAEEANRQ**LVLTFYDR^50 ◑^ ^138^IVEH**WDVIQPVPETSANR**^155 ◑^
BCAM2181 *	HP	4	2	85.9	^150^VEALL**ADESTAAR**^162 ◑^
BCAM2603 *	HP	15	4	56.0	^19^YD**GLTALNAYDEDGR**^33 ○^ ^37^YAIT**EGPYAGAK**^48 ◑^ ^73^ATVVHID**DFAAGTSR**^87 ◑^
BCAM2686 *	HP	8	3	90.9	-
BCAS0151 *	HP	14	3	90.9	-
BCAS0750	HP	28	3	90.9	-

^1^ *Burkholderia* Genome Database. ^2^ Data retrieved from LC-MS/MS analysis using Proteome Discoverer Program. PSM—Peptide Spectrum Matches. ^3^ Vaxign-ML score is the percentile rank score from the final Machine Learning classification model—recommended threshold: 90.0. ^4^ Peptides identified by LC-MS/MS were analyzed for B-cell linear epitopes using http://tools.iedb.org/bcell/ (accessed at 29 May 2023). Threshold of 0.5. B-cell epitopes shorter than 5 or larger than 25 amino acids were not considered. * Subcellular classification of outer membrane using LocTree3. ^#^ Protein with multiple localization sites. ^○^ Only found under aerobic conditions. ^●^ Only found under microaerophilic conditions. ^◑^ Found under aerobic and microaerophilic conditions.

**Table 3:** Summary of *B. cenocepacia* J2315’s selected surface-associated proteins using the surface-shaving strategy followed by LC-MS/MS. The numbers of unique peptides identified, Vaxign-ML score, and identified protein moieties containing predicted immunogenic epitopes (in bold) are shown.

ORF ^1^	Product ^1^	MW (kDa) ^1^	PSMs ^2^	Unique Peptides ^2^	Vaxign-ML Score ^3^	Surface-Exposed Protein Moieties with Predicted B Cell Linear Epitopes ^4^
BCAL1985	Putative exported isomerase	28.6	26	6	90.9	^77^E**GIPNRPD**VK^86 ◑^ ^235^AQIAQ**QLVQQKLQAFEEG**LR^254 ◑^
BCAL2645	Putative OmpA family membrane protein	21.5	22	3	90.9	^79^LAPSAAQTG**TQVTEQ**PDGSLK^99 ●^ ^178^LSAQG**MGASNPIADNATEAGR**^198 ◑^
BCAM1931	Putative porin	37.5	62	10	92.5	^44^**SLWSMGSGID**QSR^56 ◑^ ^61^GS**EDLGGG**LK^70 ◑^ ^199^LGAAYS**QANLGDGTNANGATNIAAQGR**^225 ◑^

^1^ *Burkholderia* Genome Database. ^2^ Data retrieved from LC-MS/MS analysis using Proteome Discoverer Program. PSM—Peptide Spectrum Matches. ^3^ Vaxign-ML score is the percentile rank score from the final Machine Learning classification model—recommended threshold: 90.0. ^4^ Peptides identified by LC-MS/MS were analyzed for B-cell linear epitopes using http://tools.iedb.org/bcell/ (accessed at 29 May 2023). Threshold of 0.5. B-cell epitopes shorter than 5 or larger than 25 amino acids were not considered. ^●^ Only found under microaerophilic conditions. ^◑^ Found under both conditions.

## Data Availability

All data is available as Supplementary Materials and also upon request to the corresponding authors.

## Supplementary Materials

The following supporting information can be downloaded at: https://www.mdpi.com/article/10.3390/vaccines12040398/s1, Supplementary File S1 including Table S1: Total proteins identified in *B. cenocepacia* J2315 using the “surface shaving” strategy followed by LC-MS/MS and analysis of the obtained protein moieties using Thermo Proteome Discoverer 2.5.0.400; Table S2: Proteins identified in *B. cenocepacia* J2315 using the “surface shaving” strategy followed by LC-MS/MS (Threshold score: ≥2 unique peptides) and predicted to be localized in the outer membrane (OM), extracellular (E), fimbrium, or flagella; Table S3: Proteins identified in *B. cenocepacia* J2315 using the “surface shaving” strategy followed by LC-MS/MS (Threshold score: ≥2 unique peptides) and predicted to be localized in the periplasm (PM), cytoplasmic membrane (CM), or cytoplasm (C); and Table S4: Proteins identified in *B. cenocepacia* J2315 using the “surface shaving” strategy followed by LC-MS/MS (Threshold score: ≥2 unique peptides) and predicted to be localized as unknown (U) by PSORTb. Supplementary File S2 including Table S5: Predicted moonlight proteins identified in *B. cenocepacia* J2315 using the “surface shaving” strategy followed by LC-MS/MS (Threshold score: ≥2 peptides); and Table S6: Predicted surface-exposed proteins identified in *B. cenocepacia* J2315 using the “surface shaving” strategy followed by LC-MS/MS (Threshold score: ≥2 peptides) and their predicted B-cell epitopes.
