# Supplementary material for: Surface-Exposed Protein Moieties of Burkholderia cenocepacia J2315 in Microaerophilic and Aerobic Conditions
Source: Vaccines (Basel). 2024 Apr 9;12(4):398. doi: 10.3390/vaccines12040398 (PMC11054960; doi:10.3390/vaccines12040398)
Supplement: Supplementary file 1 [file vaccines-12-00398-s001.zip › Supplementary_2.pdf]

*Supplementary materials_File 2*

*Article*

# Surface-exposed protein moieties of *Burkholderia cenocepacia* J2315 in microaerophilic and aerobic conditions

**António M.M. Seixas[1, 2], Carolina Silva[1, 2], Patrícia Mateus[1, 2], Manuel J. Rodríguez-Ortega[3], Joana R. Feliciano[1, 2], Jorge H. Leitão[1, 2*], Sílvia A. Sousa[1, 2*]**

1 Department of Bioengineering, IBB—Institute for Bioengineering and Biosciences, Instituto Superior Técnico, Universidade de Lisboa, Av. Rovisco Pais, 1049-001 Lisboa, Portugal
2 Associate Laboratory, i4HB—Institute for Health and Bioeconomy at Instituto Superior Técnico, Universidade de Lisboa, Av. Rovisco Pais, 1049-001 Lisboa, Portugal
3 Departamento de Bioquímica y Biología Molecular, Universidad de Córdoba, and Campus de Excelencia Internacional CeiA3, 14071 Córdoba, Spain
* Correspondence: Silvia A. Sousa, E-mail: sousasilvia@tecnico.ulisboa.pt; Jorge H. Leitão, E-mail: jorgeleitao@tecnico.ulisboa.pt. iBB - Institute for Bioengineering and Biosciences, Department of Bioengineering, Instituto Superior Técnico, Universidade de Lisboa, Lisboa, Portugal. Telephone: +351-218417688; Fax: +351-218419199

**Table S5 –** Predicted moonlight proteins identified in *B. cenocepacia* J2315 by using the "surface shaving" strategy followed by LC-MS/MS (Threshold score: ≥2 peptides).

| ORF | Description | Canonical Function | Moonlight Function in other bacteria[2] | PSM[1] | Peptides[1] | References |
|---|---|---|---|---|---|---|
| **BCAL0201** | N-acetyl-gamma-glutamyl-phosphate reductase (ArgC) | Cytoplasm: Catalyzes the conversion of N-acetyl-L-glutamate to N-acetyl-L-glutamate 5-phosphate (EC 1.2.1.38). | Cell surface: Binds mucus and Caco-2 cells, and human ABO blood group antigens (*Lactobacillus plantarum*). | 34 | 7 | [1] |
| **BCAL0219/ BCAL0232*** | Elongation factor Tu (TufA) | Cytoplasm: Elongation factor in protein synthesis. | Cell surface: Attachment to human cells and mucins (*Lactobacillus johnsonii*). Fibronectin binding (*Mycoplasma pneumoniae*). Factor H and plasminogen binding (*Pseudomonas aeruginosa*). | 76 | 10 | [2][3][4] |
| **BCAL0226** | DNA-directed RNA polymerase subunit beta (RpoB) | Cytoplasm: Beta subunit of DNA-directed RNA polymerase (EC 2.7.7.6) | Cell surface: Salivary mucin binding protein Muc7 (*Streptococcus gordonii*). | 15 | 7 | [5] |
| **BCAL0231** | Elongation factor G (FusA) | Cytoplasm: Elongation factor in translation. | Cell surface: Binds salivary mucin MUC7(*S. gordonii*) | 38 | 14 | [5] |
| **BCAL0739** | Phosphoglyceromutase (GpmA) | Cytoplasm: Phosphoglycerate mutase (EC 5.4.2.11) | Cell surface: Plasminogen binding (*Bifidobacterium lactis*, *Bifidobacterium bifidum*, *Bifidobacterium longum*). | 15 | 5 | [6] |
| **(BCAL1001/ BCAL2869)*/ BCAL2829** | Serine endoprotease DegP-like (MucD2/ MucD1/ HtrA) | Periplasm: Serine endoprotease (EC 3.4.21.107). | Extracellular: Cleaves E-cadherin to disrupt intercellular adhesion (*Helicobacter pylori*). | 71 / 28 | 18 / 7 | [7] |
| **BCAL1070/ BCAL3192** | Peroxiredoxin, AhpC-type | Cytoplasm: Cell redox homeostasis (EC 1.11.1.24). | Cell surface: Binds plasminogen (*Neisseria meningitidis*). | 17 / 42 | 6 / 8 | [8] |
| **BCAL1919** | Heat shock protein ClpB | Cytoplasm: Part of a stress-induced multi-chaperone system, it is involved in the recovery of the cell from heat-induced damage, in cooperation with DnaK, DnaJ and GrpE. | Extracellular: Interact with host macrophages and mediates inflammatory immune response (*Mycobacterium tuberculosis*). | 4 | 2 | [9] |

| | | | | | | |
|---|---|---|---|---|---|---|
| **BCAL1990** | Glucose-6-phosphate isomerase | Cytoplasm:<br>Catalyzes interconversion of glucose-6-phosphate and fructose-6-phosphate in glycolysis and gluconeogenesis (EC 5.3.1.9). | Cell Surface:<br>Laminin, collagen I binding (*Lactobacillus crispatus*). | 15 | 8 | [10] |
| **BCAL1996** | ATP-dependent Clp protease proteolytic subunit (ClpP) | Cytoplasm:<br>Hydrolysis of proteins to small peptides in the presence of ATP and magnesium (EC 3.4.21.92). | Cell Surface:<br>Binding of plasmin(ogen), fibronectin and binding and invasion to epithelial and endothelial cells (*Streptococcus pneumoniae*). | 25 | 5 | [11] |
| **BCAL2013** | AhpC/TSA family protein | Cytoplasm:<br>Hydroxyperoxidase activity, antioxidant (EC 1.11.1.-). | Cell surface:<br>Plasminogen binding (*N. meningitidis*). | 29 | 6 | [8] |
| **BCAL2122** | Malate synthase (AceB) | Cytoplasm:<br>Catalyzes the condensation and subsequent hydrolysis of acetyl-CoA and glyoxylate to form malate and CoA (EC 2.3.3.9) | Cell surface:<br>Binds fibronectin, laminin and A549 lung epithelial cells (*M. tuberculosis*). | 4 | 2 | [12] |
| **BCAL2152/ BCAL2153/ BCAM0088** | Peptidyl-prolyl cis-trans isomerase (PpiA/ PpiB) | Periplasm / Cytoplasm:<br>Catalyzes the cis-trans isomerization of proline imidic peptide bonds in oligopeptides (EC 5.2.1.8) | Extracellular:<br>Induces gastric epithelial cell apoptosis in a TLR4-dependent manner (*H. pylori*). | 92 / 24 /14 | 17 / 6 / 4 | [13] |
| **BCAL2179** | Enolase | Cytoplasmic Membrane:<br>Catalyzes the reversible conversion of 2-phosphoglycerate into phosphoenolpyruvate. (EC. 4.2.1.11) | Cell surface:<br>Plasminogen binding (*Aeromonas hydrophila*, *Borrelia burgdorferi*, *N. meningitidis*). | 37 | 7 | [14] |
| **BCAL2224** | Glutamine synthetase (GlnA) | Cytoplasm:<br>Synthesis of glutamine (EC 6.3.1.2) | Cell surface:<br>Binds fibronectin (*M. tuberculosis*).<br>Binding laminin (*L. crispatus*).<br>Plasminogen binding (*M. tuberculosis; L. crispatus; B. lactis*).<br>Binds collagen I and other bacterial species (*L. crispatus*). | 10 | 2 | [10][15][6] |
| **BCAL2346** | Triosephosphate isomerase (TpiA) | Cytoplasm:<br>Catalyzes the reversible interconversion of dihydroxyacetone phosphate and D-glyceraldehyde 3-phosphate (glycolysis) (EC 5.3.1.1). | Cell surface:<br>Plasminogen binding (*Streptococcus anginosus, Streptococcus oralis*).<br>Adhesin, contact-mediated killing of Cryptococcus (*Staphylococcus aureus*). | 9 | 4 | [16][17] |

| | | | | | | |
|---|---|---|---|---|---|---|
| **BCAL2433** | Transaldolase B (TalB) | Cytoplasm: Important for the balance of metabolites in the pentose-phosphate pathway (EC 2.2.1.2) | Extracellular: Binds mucin (*B. longum*). | 62 | 13 | [18] |
| **BCAL2730** | Putative ATP-dependent Clp protease ATP-binding subunit (ClpA) | Cytoplasm: Protease ATP-dependent protein. Chymotrypsin-like activity. Major role in the degradation of misfolded proteins. | Cell surface: Binding of plasminogen and fibronectin. Adhesion and invasion of epithelial and endothelial cells (*S. pneumoniae*). | 12 | 4 | [11] |
| **BCAL2757** | Superoxide dismutase (SodB) | Periplasm: Superoxide dismutase (EC 1.15.1.1) | Cell surface: Adhesin binding mucus-associated proteins. Binding to epithelial cell aldolase, GAPDH and cyclophilin A (*Mycobacterium avium*). | 42 | 5 | [19] |
| **BCAL2767** | Ornithine carbamoyltransferase (ArgF) | Cytoplasm: Reversibly catalyzes the transfer of the carbamoyl group from carbamoyl phosphate to the N(epsilon) atom of ornithine to produce L-citrulline (EC 2.1.3.3). | Cell surface: Binds fibronectin (*Staphylococcus epidermidis*). | 11 | 2 | [20] |
| **BCAL2777** | N-acetylmuramoyl-L-alanine amidase | Periplasm: Autolysin that hydrolyzes the amide bond between N-acetylmuramoyl and L-amino acids in certain cell wall glycopeptides (EC 3.5.1.28). | Extracellular: Fibrinogen and fibronectin binding (*S. aureus*). | 18 | 6 | [21] |
| **BCAL2839** | Fructose-1,6-bisphosphate aldolase (CbbA) | Cytoplasm: Glycolysis and gluconeogenesis (EC 4.1.2.13). | Cell surface: Adhesion to host (*N. meningitidis*). Binds to host Flamingo cadherin receptor (FCR) (*S. pneumoniae*). | 30 | 7 | [22][23] |
| **BCAL2841** | Phosphoglycerate kinase | Cytoplasm: Phosphoglycerate kinase (EC 2.7.2.3). | Cell surface: Plasminogen binding (*S. anginosus, S. oralis, S. pneumoniae*). Cell surface virulence factor (*A. hydrophila*). | 60 | 11 | [16][24][25] |
| **BCAL3004** | Chorismate mutase | Periplasm: Key enzyme in the shikimate pathway (EC 5.4.99.5). | Extracellular: Involved in virulence, being important in bacterium-rice interactions (*Xanthomonas oryzae*). | 8 | 3 | [26] |

| | | | | | | |
|---|---|---|---|---|---|---|
| **BCAL3146** | Chaperonin GroEL | Cytoplasm: Prevents misfolding and promotes the refolding and proper assembly of unfolded polypeptides generated under stress conditions. | Extracellular: Adhesion to host (*Salmonella typhimurium*). | 241 | 27 | [27] |
| **BCAL3270** | Molecular chaperone DnaK | Cytoplasm: Chaperone | Cell surface: Binds plasminogen (*N. meningitidis*). | 23 | 6 | [8] |
| **BCAL3272** | Heat shock protein GrpE | Cytoplasm: Part of the HSP70 chaperone complex (GrpE, DnaJ, and DnaK), which is important for protein folding and helps to protect cells from stress. | Cell surface: Interaction with Salivary Proline-rich Proteins of Pharyngeal Epithelial Cell (*Streptococcus pyogenes*) | 11 | 4 | [28] |
| **BCAL3388** | Glyceraldehyde-3-phosphate (G-3-P) dehydrogenase 1 (GapA) | Cytoplasm: Glycolytic enzyme that catalyses the conversion of G-3-P to 1, 3-bisphosphoglycerate (EC 1.2.1.-). | Cell surface: Binds plasminogen and Fibrinogen (*Escherichia coli; Francisella tularensis;* ). Binds fibronectin (*F. tularensis*) | 84 | 13 | [29] |
| **BCAL3453** | Preprotein translocase subunit SecA | Cytoplasm: Part of the Sec protein translocase complex. | Cell surface: Binds salivary mucin (S. gordonii) | 22 | 6 | [5] |
| **BCAM0941** | 6-phosphogluconate dehydrogenase (6PGD) | Cytoplasm: Produces NADPH by converting 6-phosphogluconate to ribulose 5-phosphate in the pentose phosphate pathway (EC 1.1.1.44). | Cell surface: Adhesin (*S. pneumoniae*). | 76 | 7 | [30] |
| **BCAM0965** | Malate dehydrogenase | Cytoplasm: Tricarboxylic acid cycle (TCA) (EC 1.1.1.37). | Cell surface: Binds fibronectin and plasminogen (*Brucella abortus*). | 88 | 16 | [31] |
| **BCAM1309** | γ-glutamyltransferase precursor 2 (Ggt2) | Periplasm: Converts glutamine into glutamate and ammonia, and converts glutathione into glutamate and cysteinylglycine (EC 2.3.2.2). | Extracellular: Causes glutamine and glutathione consumption in the host cells. Inhibition of T cell-mediated immunity and dendritic cell differentiation (*H. pylori*) | 66 | 17 | [32] |
| **BCAM2821** | Malate synthase G (GlcB) | Cytoplasm: Malate synthase, glyoxylate cycle (EC 2.3.3.9). | Cell surface: Binds fibronectin, laminin and A549 lung epithelial cells (*M. tuberculosis*). | 9 | 4 | [12] |

[1] Peptides identified using surface-shaving with trypsin and LC-MS/MS analysis.

[2] "Moonlight proteins" in other bacteria with secondary function at the Outer membrane or Extracellular. Information retrieved from Database MoonProt 3.0 and MultitaskProtDBII.

* In-paralogs.

## References


1. Kinoshita H, Uchida H, Kawai Y, et al. Cell surface *Lactobacillus plantarum* LA 318 glyceraldehyde-3-phosphate dehydrogenase (GAPDH) adheres to human colonic mucin. *J Appl Microbiol*. **2008**;104(6):1667-1674. doi:10.1111/j.1365-2672.2007.03679.x
2. Granato D, Bergonzelli GE, Pridmore RD, Marvin L, Rouvet M, Corthésy-Theulaz IE. Cell Surface-Associated Elongation Factor Tu Mediates the Attachment of *Lactobacillus johnsonii* NCC533 (La1) to Human Intestinal Cells and Mucins. *Infect Immun*. **2004**;72(4):2160-2169. doi:10.1128/IAI.72.4.2160-2169.2004
3. Dallo SF, Kannan TR, Blaylock MW, Baseman JB. Elongation factor Tu and E1 β subunit of pyruvate dehydrogenase complex act as fibronectin binding proteins in *Mycoplasma pneumoniae*. *Mol Microbiol*. **2002**;46(4):1041-1051. doi:10.1046/j.1365-2958.2002.03207.x
4. Kunert A, Losse J, Gruszin C, et al. Immune Evasion of the Human Pathogen *Pseudomonas aeruginosa* : Elongation Factor Tuf Is a Factor H and Plasminogen Binding Protein. *J Immunol*. **2007**;179(5):2979-2988. doi:10.4049/jimmunol.179.5.2979
5. Kesimer M, Kiliç N, Mehrotra R, Thornton DJ, Sheehan JK. Identification of salivary mucin MUC7 binding proteins from *Streptococcus gordonii*. *BMC Microbiol*. **2009**;9(1):163. doi:10.1186/1471-2180-9-163
6. Candela M, Bergmann S, Vici M, et al. Binding of human plasminogen to *Bifidobacterium*. *J Bacteriol*. **2007**;189(16):5929-5936. doi:10.1128/JB.00159-07
7. Hoy B, Löwer M, Weydig C, et al. *Helicobacter pylori* HtrA is a new secreted virulence factor that cleaves E-cadherin to disrupt intercellular adhesion. *EMBO Rep*. **2010**;11(10):798-804. doi:10.1038/embor.2010.114
8. Knaust A, Weber MVR, Hammerschmidt S, Bergmann S, Frosch M, Kurzai O. Cytosolic Proteins Contribute to Surface Plasminogen Recruitment of *Neisseria meningitidis*. *J Bacteriol*. **2007**;189(8):3246-3255. doi:10.1128/JB.01966-06
9. Kędzierska-Mieszkowska S, Zolkiewski M. Hsp100 Molecular Chaperone ClpB and Its Role in Virulence of Bacterial Pathogens. *Int J Mol Sci*. **2021**;22(10):5319. doi:10.3390/ijms22105319
10. Kainulainen V, Loimaranta V, Pekkala A, et al. Glutamine Synthetase and Glucose-6-Phosphate Isomerase Are Adhesive Moonlighting Proteins of *Lactobacillus crispatus* Released by Epithelial Cathelicidin LL-37. *J Bacteriol*. **2012**;194(10):2509-2519. doi:10.1128/JB.06704-11
11. Agarwal V, Kuchipudi A, Fulde M, Riesbeck K, Bergmann S, Blom AM. *Streptococcus pneumoniae* Endopeptidase O (PepO) Is a Multifunctional Plasminogen- and Fibronectin-binding Protein, Facilitating Evasion of Innate Immunity and Invasion of Host Cells. *J Biol Chem*. **2013**;288(10):6849-6863. doi:10.1074/jbc.M112.405530
12. Kinhikar AG, Vargas D, Li H, et al. *Mycobacterium tuberculosis* malate synthase is a laminin-binding adhesin. *Mol Microbiol*. **2006**;60(4):999-1013. doi:10.1111/j.1365-2958.2006.05151.x
13. Henderson B, Martin A. Bacterial Virulence in the Moonlight: Multitasking Bacterial Moonlighting Proteins Are Virulence Determinants in Infectious Disease. *Infect Immun*. **2011**;79(9):3476-3491. doi:10.1128/IAI.00179-11
14. Sha J, Erova TE, Alyea RA, et al. Surface-Expressed Enolase Contributes to the Pathogenesis of Clinical Isolate SSU of *Aeromonas hydrophila*. *J Bacteriol*. **2009**;191(9):3095-3107. doi:10.1128/JB.00005-09
15. Xolalpa W, Vallecillo AJ, Lara M, et al. Identification of novel bacterial plasminogen-binding proteins in the human pathogen *Mycobacterium tuberculosis* . *Proteomics*. **2007**;7(18):3332-3341. doi:10.1002/pmic.200600876
16. Kinnby B, Booth NA, Svensäter G. Plasminogen binding by oral streptococci from dental plaque and inflammatory lesions. *Microbiology*. **2008**;154(3):924-931. doi:10.1099/mic.0.2007/013235-0
17. Yamaguchi M, Ikeda R, Nishimura M, Kawamoto S. Localization by scanning immunoelectron microscopy of triosephosphate isomerase, the molecules responsible for contact-mediated killing of Cryptococcus, on the surface of *Staphylococcus*. *Microbiol Immunol*. **2010**;54(6):368-370. doi:10.1111/j.1348-0421.2010.00225.x
18. Nishiyama K, Takaki T, Sugiyama M, et al. Extracellular Vesicles Produced by *Bifidobacterium longum* Export Mucin-Binding Proteins. *Appl Environ Microbiol*. **2020**;86(19). doi:10.1128/AEM.01464-20

19. Reddy VM, Suleman FG. *Mycobacterium avium*-superoxide dismutase binds to epithelial cell aldolase, glyceraldehyde-3-phosphate dehydrogenase and cyclophilin A. *Microb Pathog*. **2004**;36(2):67-74. doi:10.1016/j.micpath.2003.09.005
20. Hussain M, Peters G, Chhatwal GS, Herrmann M. A Lithium Chloride-Extracted, Broad-Spectrum-Adhesive 42-Kilodalton Protein of *Staphylococcus epidermidis* Is Ornithine Carbamoyltransferase. *Infect Immun*. **1999**;67(12):6688-6690. doi:10.1128/IAI.67.12.6688-6690.1999
21. Heilmann C, Hartleib J, Hussain MS, Peters G. The Multifunctional *Staphylococcus aureus* Autolysin Aaa Mediates Adherence to Immobilized Fibrinogen and Fibronectin. *Infect Immun*. **2005**;73(8):4793-4802. doi:10.1128/IAI.73.8.4793-4802.2005
22. Tunio SA, Oldfield NJ, Berry A, Ala'Aldeen DAA, Wooldridge KG, Turner DPJ. The moonlighting protein fructose-1, 6-bisphosphate aldolase of *Neisseria meningitidis*: surface localization and role in host cell adhesion. *Mol Microbiol*. **2010**;76(3):605-615. doi:10.1111/j.1365-2958.2010.07098.x
23. Blau K, Portnoi M, Shagan M, et al. Flamingo Cadherin: A Putative Host Receptor for *Streptococcus pneumoniae*. *J Infect Dis*. **2007**;195(12):1828-1837. doi:10.1086/518038
24. Fulde M, Bernardo-García N, Rohde M, et al. *Pneumococcal* phosphoglycerate kinase interacts with plasminogen and its tissue activator. *Thromb Haemost*. **2014**;112(03):401-416. doi:10.1160/TH13-05-0421
25. Henderson B, Martin A. Bacterial Moonlighting Proteins and Bacterial Virulence. In: ; 2011:155-213. doi:10.1007/82_2011_188
26. Degrassi G, Devescovi G, Bigirimana J, Venturi V. *Xanthomonas oryzae* pv. *oryzae* XKK.12 Contains an AroQ $_\gamma$ Chorismate Mutase That Is Involved in Rice Virulence. *Phytopathology®*. **2010**;100(3):262-270. doi:10.1094/PHYTO-100-3-0262
27. Ensgraber M, Loos M. A 66-kilodalton heat shock protein of *Salmonella typhimurium* is responsible for binding of the bacterium to intestinal mucus. *Infect Immun*. **1992**;60(8):3072-3078. doi:10.1128/iai.60.8.3072-3078.1992
28. Murakami J, Terao Y, Morisaki I, Hamada S, Kawabata S. Group A *Streptococcus* Adheres to Pharyngeal Epithelial Cells with Salivary Proline-rich Proteins via GrpE Chaperone Protein. *J Biol Chem*. **2012**;287(26):22266-22275. doi:10.1074/jbc.M112.350082
29. Kopeckova M, Pavkova I, Stulik J. Diverse Localization and Protein Binding Abilities of Glyceraldehyde-3-Phosphate Dehydrogenase in Pathogenic Bacteria: The Key to its Multifunctionality? *Front Cell Infect Microbiol*. **2020**;10. doi:10.3389/fcimb.2020.00089
30. Amblee V, Jeffery CJ. Physical Features of Intracellular Proteins that Moonlight on the Cell Surface. *PLoS One*. **2015**;10(6):e0130575. doi:10.1371/journal.pone.0130575
31. Han X, Tong Y, Tian M, et al. Characterization of the immunogenicity and pathogenicity of malate dehydrogenase in *Brucella abortus*. *World J Microbiol Biotechnol*. **2014**;30(7):2063-2070. doi:10.1007/s11274-014-1631-2
32. Ricci V. *Helicobacter pylori* gamma-glutamyl transpeptidase and its pathogenic role. *World J Gastroenterol*. **2014**;20(3):630. doi:10.3748/wjg.v20.i3.630

**Table S6 –** Predicted surface-exposed proteins identified in *B. cenocepacia* J2315 by using the “surface shaving” strategy followed by LC-MS/MS (Threshold score: ≥2 peptides) and their predicted B cell epitopes. HP – hypothetical protein.

| ORF (Description) | Peptides identified[1] | | | B cell epitopes average[2] | Predicted B cell epitopes[2,3] |
|---|---|---|---|---|---|
| | Aerobic conditions | Microaerophilic condition | Both conditions | | |
| BCAL0076 (Putative lipoprotein) | | | $^{86}$ QEANDMSAQHNGGLSGDEQR $^{105}$<br>$^{106}$ VLNQELDTAAR $^{116}$ | 0.510 | $^{85}$R**QEANDMSAQHNGGLSGDEQR**VLNQ$^{109}$ |
| BCAL0151 (Extracellular ligand binding protein) | $^{33}$IGHVAPLTGGIAHLGK$^{48}$<br>$^{96}$LVDDKVVAVVGHLNSGTSIPASK$^{118}$<br>$^{199}$VMSHDATNDKAVDFR$^{213}$<br>$^{355}$ GDLQHGVISLYNYK $^{368}$ | | $^{56}$ LAVEEINAK $^{64}$<br>$^{73}$ ITLQLDPQDDAADPR $^{87}$<br>$^{73}$ ITLQLDPQDDAADPRQATQVAQK $^{95}$<br>$^{101}$ VVAVVGHLNSGTSIPASK $^{118}$<br>$^{119}$ IYSDAGVVQISPSATNPAYTQQGFK $^{143}$<br>$^{148}$ VVATDAQQGPALADYAHSK $^{166}$<br>$^{170}$ SVAVVDDS $^{177}$<br>$^{170}$ SVAVVDDSTAYGQGLANEFEK $^{190}$<br>$^{219}$ IKGENPDAIMYGGMDATGGPFAK $^{241}$<br>$^{286}$ MPGGAAFK $^{293}$<br>$^{325}$ ANSTDPAKILAAMPATK $^{341}$<br>$^{333}$ ILAAMPATK $^{341}$<br>$^{342}$ YTGVIGTTTFDSK $^{354}$<br>$^{373}$ SLLDEVK $^{379}$ | 0.439 | $^{64}$ **K**GLTIGGQK $^{72}$<br>$^{82}$ **DAADPRQATQV** $^{92}$<br>$^{136}$ **AYTQQG** $^{141}$<br>$^{202}$ **HDATNDKAVD** $^{211}$<br>$^{262}$ KLADLAGD $^{269}$<br>$^{282}$ SLEK**MPGG** $^{289}$<br>$^{297}$ EKRFGQP $^{303}$<br>$^{326}$ **NSTDPAK** $^{332}$<br>$^{353}$ **SKGDLQ** $^{358}$<br>$^{372}$ K**SLLD** $^{376}$ |
| BCAL0304 (VacJ-like lipoprotein) | $^{195}$ANLLGAGDVLDAAALDK$^{211}$ | | $^{232}$GEAAVTSNNDALPK$^{245}$ | 0.469 | $^{27}$ VQTPTKGDPLE $^{37}$<br>$^{125}$ KLPKHTADF $^{133}$<br>$^{175}$ YVKPDG $^{180}$<br>$^{194}$ R**ANLLGAGDV** $^{203}$ |
| BCAL0343 (Putative type VI secretion system protein -TssD) | | $^{30}$ SWDHSIVQPR $^{39}$<br>$^{99}$ VQYLEVK $^{105}$<br>$^{144}$IGGNQGGNTQGAWSLTK$^{160}$ | $^{40}$ SATASTAGGHTMTR $^{53}$ | 0.503 | $^{13}$ VKGESADKDHQ $^{23}$<br>$^{31}$**WDHSIVQPRSATASTAGGHTMTR**C $^{54}$<br>$^{92}$ ADGEGKR**V** $^{99}$<br>$^{116}$ PSVREEGLP $^{124}$ |
| BCAL0349 | $^{195}$ VSASEQGVLDQTLAN | | | 0.516 | $^{5}$ IQVRRAR $^{11}$ |

| | | | | | |
|---|---|---|---|---|---|
| (Type VI secretion system-associated protein TagL) | R $^{210}$<br>$^{257}$ TSNIALSQAR $^{266}$ | | | | $^{40}$ GATVTPVGN $^{48}$<br>$^{184}$ NPTYTIKNGLR**VSASEQGVL** $^{203}$<br>$^{213}$ EFETGSATLTPQ $^{224}$<br>$^{251}$ DNSGNR**TSNIAL** $^{262}$<br>$^{290}$ GPDQPIAPNDTADGRARN $^{307}$ |
| BCAL0360 (HP) | | $^{48}$ DNAPLDER $^{55}$<br>$^{72}$AANHQVIGTSETYSSVQAR$^{90}$ | $^{21}$ SANGEIILASER $^{32}$ | 0.488 | $^{8}$ KKATNGQ $^{14}$<br>$^{31}$ **ER**YEEKSG $^{38}$<br>$^{49}$ **NAPLDER**YERKLAHN $^{63}$<br>$^{80}$ **TSETYSSVQA** $^{89}$<br>$^{99}$ RDAPIAET $^{106}$ |
| BCAL0389 (Thiol:disulfide interchange protein - DsbC) | $^{224}$RLPGAVSADQLNQALASSK$^{242}$ | | $^{40}$ LGNDAPIK $^{47}$ | 0.481 | $^{24}$ QADQTTDK $^{31}$<br>$^{33}$ KATLQAR**LGNDAPIK**SVSKSPVA $^{55}$<br>$^{223}$ R**RLPGAVSADQL** $^{234}$ |
| BCAL0562 (Flagellin synthesis anti-sigma-28 factor - FglM) | | $^{22}$APSGTAQSSAQAGDAGSTGGDTTVNLSGLSGQLR$^{55}$<br>$^{89}$ IADGVLNTAR $^{98}$ | $^{3}$ IDSTPKPSPLAPTGNGAAR $^{21}$ | 0.553 | $^{51}$ **SGQLR**SVSASGDADIDTG $^{68}$<br>$^{99}$ ELLQRQR $^{105}$ |
| BCAL0565 (Flagellar basal body rod protein FlgC) | | $^{47}$ QVVFATDPMGGAR $^{59}$<br>$^{60}$ TASGQGVGGVR $^{70}$<br>$^{118}$ SYQANVETLNTAK $^{130}$ | | 0.485 | $^{32}$ ADSATGPDGKPY $^{43}$<br>$^{54}$ **PMGGARTASGQGVGGV** $^{69}$ |
| BCAL0577 (Flagellar hook-associated protein - FlgL) | | $^{108}$ SALSTQLQGYR $^{118}$<br>$^{141}$TATAPFSNAPGGGVTYSGDTGSR$^{163}$ | $^{164}$ EVQIADTR $^{171}$ | 0.535 | $^{17}$ SDQQAQLSQLYQQISSGVSLATPA $^{40}$<br>$^{98}$ GDGSLSDSNR**SA** $^{109}$<br>$^{403}$ LSLFQ $^{407}$ |
| BCAL0849 (Metallo peptidase, subfamily M48B) | | $^{147}$AMQTAYAVSAAR$^{158}$<br>$^{159}$SAAGAASPGVAALSSSQLGDITEK$^{182}$ | | 0.469 | $^{23}$ QSLDANSLTSAGT $^{35}$<br>$^{39}$ KAATLSDSD $^{47}$<br>$^{60}$ DAESKIAPANSAYAKR $^{75}$<br>$^{85}$ DMTLNGQK $^{92}$<br>$^{172}$ **SSSQLGDIT** $^{180}$<br>$^{206}$ QKGMSQK $^{212}$<br>$^{223}$ QMDGGQSSMMSSHPSSASRAQ $^{243}$ |

| | | | | |
|---|---|---|---|---|
| BCAL1105 (HP) | | $^{37}$ DAMGHDAMAK $^{46}$<br>$^{63}$ DAMGHDAMK $^{71}$ | 0.504 | $^{23}$ QNDAMSKDGMSMSK**DAMGH** $^{41}$ |
| BCAL1390 (Glucanase) | $^{267}$ ADPLAAPLLAK $^{277}$<br>$^{365}$ FGADGTLDTR $^{374}$ | | 0.461 | $^{17}$ TGAADAAGAGCSVAWPRWDA $^{36}$<br>$^{88}$ NNLAQGDLSAH $^{98}$<br>$^{107}$ APDGAWRVLDA $^{117}$<br>$^{138}$ WRERSY $^{143}$<br>$^{158}$ ETATVPG $^{164}$<br>$^{175}$ GFKLADG $^{181}$<br>$^{203}$ PDDRR $^{207}$<br>$^{234}$ YRAGKGFGPDPDTHA $^{248}$<br>$^{267}$ **ADPLA** $^{271}$<br>$^{289}$ GAPPERVDTTTGVAGPND $^{306}$<br>$^{325}$ HALADA $^{330}$<br>$^{332}$ AARVDTLARQSAP $^{344}$<br>$^{370}$ **TLDTR**WGDRS $^{379}$ |
| BCAL1848 (HP) | | $^{98}$ APQALVVTTR $^{107}$<br>$^{108}$ SAGSGGYVGAQAYVTTSR $^{125}$ | 0.489 | $^{21}$ AADECGFVKKVELPSR $^{36}$<br>$^{45}$ GALEPCSTGS $^{54}$<br>$^{60}$ YSTAHAAPGFDTDDY $^{74}$<br>$^{91}$ TADLGAR**AP** $^{99}$<br>$^{107}$ **RSAGSGGYVG** $^{116}$<br>$^{132}$ SVDGLAPDVD $^{141}$ |
| BCAL1849 (HP) | | $^{72}$ GGGTGQLEYTVK $^{83}$<br>$^{88}$ TFGNTQETR $^{96}$<br>$^{153}$ GVANVQLSFQAAAPK $^{167}$ | 0.520 | $^{61}$ FAAPPVKGSLK**GGGTGQ** $^{77}$<br>$^{143}$ VRLAPSVDAK**GVA** $^{155}$<br>$^{164}$**AAPK**GTRSVTAGGKSLQCPDVVS VS$^{188}$<br>$^{198}$ NGGSKSVTM $^{206}$ |
| BCAL1893 (Family M23 peptidase) | $^{201}$ GVNIGGTAGEAVK $^{213}$ | $^{85}$ IALENGQNYR $^{94}$<br>$^{229}$ GYGNLIIIK $^{237}$ | 0.523 | $^{5}$ SMLRAMQNNRSREPLTLA $^{22}$<br>$^{191}$ LNGFDDAK $^{198}$<br>$^{251}$ ALMVKEGDAVTK $^{262}$<br>$^{287}$ GKPVDPLKY $^{295}$ |
| BCAL1938 (Cysteine peptidase, family C40) | $^{346}$ TSTADDPIAR $^{355}$ | $^{324}$ SIEPAPVQVLR $^{334}$ | 0.532 | $^{109}$ GRGVSIEPD $^{117}$<br>$^{161}$ LTNPYWAK $^{168}$<br>$^{334}$ **R**ASTQSAPVSPR**TSTADDPIAR**FA $^{357}$ |

| | | | | | |
|---|---|---|---|---|---|
| BCAL1961<br>(HP) | $^{82}$ VAAAIATTPNVDLEK $^{96}$<br>$^{174}$GNHASTVTLLLDQGADPQV<br>K$^{193}$ | | $^{43}$ FDDIADIGK $^{51}$<br>$^{58}$ LDPNTLAPNGDPILVIAAR $^{76}$<br>$^{82}$ VAAAIATTPNVDLEKEDK $^{99}$<br>$^{131}$ GWAPLHYAATNGQDAVVK $^{148}$<br>$^{194}$ NQLGITALEFAK $^{205}$<br>$^{206}$ HYNAPDAIEILSK $^{218}$<br>$^{223}$ IGASTPADAQK $^{233}$ | 0.472 | $^{58}$ **LDPNTLAPNG** $^{67}$<br>$^{92}$ **VDLEKEDK**AG $^{101}$<br>$^{125}$ AEVSKK**G** $^{131}$<br>$^{155}$ AYIDTASPN $^{163}$<br>$^{187}$ **GADPQVKNQLGIT** $^{199}$<br>$^{219}$ RTTR**IGASTPADAQK** $^{233}$ |
| BCAL1985<br>(Putative exported<br>isomerase) | | | $^{68}$ EILMQEAIR $^{76}$<br>$^{77}$ EGIPNRPDVK $^{86}$<br>$^{87}$ AQVAVAQQTVVLR $^{99}$<br>$^{186}$ AYVPEFAAAAQK $^{197}$<br>$^{235}$ AQIAQQLVQQK $^{245}$<br>$^{246}$ LQAFEEGLR $^{254}$ | 0.494 | $^{45}$LVQQGQTDGPQLQQAVRQELVN<br>RE$^{68}$<br>$^{78}$ **GIPNRPD** $^{84}$<br>$^{198}$ LQKGQMTDT $^{206}$<br>$^{221}$ DIRDIAPPPF $^{230}$<br>$^{240}$ **QLVQQKLQAFEEG** $^{252}$ |
| BCAL2022<br>(PspA/IM30 family<br>protein) | | | $^{13}$ GLLNDAADSVQDPSR $^{27}$<br>$^{35}$ ELDDSIGR $^{42}$<br>$^{78}$ ALQGGDEALAR $^{88}$<br>$^{150}$ DVAASALGGIGGK $^{162}$<br>$^{163}$ NLSEDFQK $^{170}$ | 0.503 | $^{20}$ **DSVQDPSR**D $^{28}$<br>$^{156}$ **LGGIGGKNLSEDFQK**LEDK $^{174}$<br>$^{215}$ AALKKQLD $^{222}$ |
| BCAL2229<br>(HP) | $^{110}$ WFAAAGLR $^{117}$<br>$^{311}$ SPHGIYFYDR $^{320}$ | | $^{46}$ EVVSTMPTGK $^{55}$<br>$^{121}$ VDIYGYDGR $^{129}$<br>$^{137}$ VPLAVMPSHLAFTK $^{150}$<br>$^{184}$ VPAGLWLTPDDK $^{195}$<br>$^{232}$ SLADGTHVAVTNR $^{244}$<br>$^{291}$ KVGIIDLASR $^{300}$<br>$^{292}$ VGIIDLASR $^{300}$<br>$^{301}$ KLVQTIAVGR $^{310}$<br>$^{302}$ LVQTIAVGR $^{310}$ | 0.427 | $^{6}$ SLGRT $^{10}$<br>$^{87}$ GKLQRTVE $^{94}$<br>$^{130}$ DLKLVKR**VPLA** $^{140}$<br>$^{174}$ TVKWKMK $^{180}$<br>$^{215}$ QKVVKQIY $^{222}$<br>$^{257}$ LTNVGDITGLLP $^{268}$<br>$^{300}$ **RKLVQ** $^{304}$ |
| BCAL2413<br>(HP) | | | $^{70}$ QMLFVDTVSASGAR $^{83}$<br>$^{97}$ EATLRDEIADPK $^{108}$<br>$^{102}$ DEIADPK $^{108}$ | 0.474 | $^{22}$ AQLTVEEIDADARQQ $^{36}$<br>$^{79}$ **ASGAR**YQAGRYTWWTKGK**EA** $^{98}$<br>$^{101}$ R**DEIADPK**SPP $^{111}$<br>$^{119}$ VEKKK $^{123}$ |
| BCAL2476<br>(HP) | | $^{5}$ YYTLSDLSTGK $^{15}$<br>$^{16}$ DLNSLDMFSSNGK $^{28}$ | $^{38}$ SYITELLSK $^{46}$<br>$^{72}$ KFIIDDNLK $^{80}$ | 0.526 | $^{61}$ SNGKPAECRER**KFIIDD** $^{77}$<br>$^{86}$ NSFSSC $^{91}$ |

| | | | | | |
|---|---|---|---|---|---|
| BCAL2645<br>(Putative OmpA family protein) | | $^{79}$LAPSAAQTGTQVTEQPDGS LK$^{99}$ | $^{161}$ AQSVVNALVQR $^{171}$<br>$^{178}$ LSAQGMGASNPIADNATEAGR $^{198}$ | 0.502 | $^{88}$ **TQVTEQ** $^{93}$<br>$^{109}$ ATNQYAITPA $^{118}$<br>$^{145}$ DSTGSAQLNQTL $^{156}$<br>$^{183}$ **MGASNPIADNATEAGR**AQN $^{201}$ |
| BCAL2820<br>(RND-4 efflux system outer membrane protein - oprM) | | $^{89}$ VSVLNIEAAR $^{98}$<br>$^{123}$ LPNSLTAVPGR $^{133}$ | $^{401}$ GTYDQQIAALER $^{412}$ | 0.519 | $^{152}$ FGRVQSLKDQAL $^{163}$<br>$^{167}$ LSTSY $^{172}$<br>$^{241}$ ANQQAQARARAQ $^{252}$<br>$^{297}$ PDVMQAEQ $^{304}$<br>$^{314}$ GAARAAFFPK $^{323}$<br>$^{329}$ AFGTASPTLGGLFKAGTA $^{346}$<br>$^{446}$ YAAQQ $^{450}$ |
| BCAL2958<br>(Putative ompA family protein) | $^{123}$IEGMNTEVVVATGYTDR$^{139}$ | | $^{56}$ DAFWTPATANAK $^{67}$<br>$^{93}$ ITYQADALFDFDK $^{105}$<br>$^{106}$ ATLKPLGK $^{113}$<br>$^{114}$ QKLDELASK $^{122}$<br>$^{116}$ LDELASK $^{122}$<br>$^{123}$ IEGMNTEVVVATGYTDR $^{139}$ *<br>$^{140}$ IGSDKYNDR $^{148}$<br>$^{203}$ RVEVEVVGTQQVQK $^{216}$<br>$^{204}$ VEVEVVGTQQVQK $^{216}$ | 0.512 | $^{101}$ **FDFDKATLKPLGKQ** $^{114}$<br>$^{139}$ **RIGSDKYNDR**L $^{149}$<br>$^{211}$ **TQQVQK**TTV $^{219}$ |
| BCAL3149<br>(HP) | | | $^{43}$ DGEHTEIR $^{50}$<br>$^{61}$ MDFVSPHR $^{68}$<br>$^{69}$ GAVLAYDPGDGK $^{80}$<br>$^{122}$ NVHALQQGGATVTEGEEAVGGR $^{143}$ | 0.483 | $^{11}$ IGASMTADPV $^{20}$<br>$^{40}$ SAR**DGEH** $^{46}$<br>$^{101}$ PLVRDRTGHRVDRSDVG $^{117}$<br>$^{124}$ **HALQQGGATVTEGEEAVGG** $^{142}$<br>$^{152}$ GAPAHAVDGV $^{161}$<br>$^{180}$ SYASDDDT $^{187}$ |
| BCAL3203<br>(Tol-Pal system protein - TolB) | | | $^{96}$ GANAFVAGSVNR $^{107}$<br>$^{176}$ YQLQISDSDGQNAR $^{189}$<br>$^{210}$ VAYVSFER $^{217}$<br>$^{326}$ VTFTGSYNTSPR $^{337}$ | 0.454 | $^{47}$ FTNEANLPQQVTS $^{59}$<br>$^{123}$ VKQQSLG $^{129}$<br>$^{150}$ YIYQKLLGVRGVF $^{162}$<br>$^{185}$ **GQNAR**IA $^{191}$<br>$^{229}$ TGRRYMVSDQKG $^{240}$<br>$^{274}$ GGLRRLTQS $^{282}$<br>$^{316}$ QGESAGAAQR**V** $^{326}$<br>$^{364}$ TGAANAITNTNRD $^{376}$ |

| | | | | | |
|---|---|---|---|---|---|
| | | | | | $^{408}$ SAPPQILSVQGGS $^{420}$ |
| BCAL3204 (Putative OmpA family lipoprotein) | | | $^{97}$ HVLIQGNTDER $^{107}$<br>$^{108}$ GTSEYNLALGQK $^{119}$<br>$^{127}$ AMALLGVNDSQMEAVSLGK $^{145}$<br>$^{163}$ RADLVYQQ $^{170}$ | 0.507 | $^{67}$ YFDFDSYSVKDEYQP $^{81}$<br>$^{105}$ **DERGTSEYNLAL** $^{116}$<br>$^{143}$ **LGK**EKPQATGHDEASWAQN $^{161}$ |
| BCAL3311 (BcnA) | | | $^{41}$ QMNVPTEGAFK $^{51}$<br>$^{64}$ AAQGSAQMTIDVASFDLGDK $^{83}$<br>$^{84}$ MYNDQVAGK $^{92}$<br>$^{99}$ TYPQATFVSSAIAPAGGNK $^{117}$<br>$^{157}$ SAFNVGTGEWK $^{167}$<br>$^{168}$ DTSIVADEVQIK $^{179}$ | 0.455 | $^{21}$ SGAAHADV $^{28}$<br>$^{78}$ **FDLGDKMYNDQVAGK**DWFDAK $^{98}$<br>$^{158}$ **AFNVGTGEWKDTSIVA** $^{173}$ |
| BCAL3394 (Putative exported ribonuclease) | $^{38}$EAVPADVAASGLDTIPTASLPR$^{59}$<br>$^{96}$ GYYHEYTVPTPR $^{107}$ | | $^{79}$ DGVVFGNR $^{86}$ | 0.509 | $^{121}$ PLRRIDN $^{127}$ |
| BCAL3426 (Putative lipoprotein - SlyB) | $^{56}$IQSDGGGSAIGTLGGGALGAVAGSAIGGGK$^{85}$ | | $^{121}$ LDNGDLR $^{127}$<br>$^{128}$ SITQAASGEAFR $^{139}$ | 0.474 | $^{25}$ APGSADVYSVGQAQREQT $^{42}$<br>$^{55}$ R**IQSDGGGSAIGTL** $^{68}$<br>$^{78}$ **GSAIGGGK**G $^{86}$<br>$^{107}$ ENLSTANG $^{114}$<br>$^{135}$ **GEAFR** $^{139}$ |
| BCAL3427 (Histone H1-like protein - HctB) | | $^{180}$KAAPATTASTASVAPASGVK$^{199}$ | $^{181}$ AAPATTASTASVAPASGVK $^{199}$ | 0.586 | - |
| BCAM0900 (HP) | $^{102}$YQMSSTPDTSPKPHDAGIPITR$^{123}$ | | $^{34}$ AEANEAAGLPDLR $^{46}$<br>$^{48}$ INRPGAEVTSK $^{58}$<br>$^{59}$ VDFADIR $^{65}$<br>$^{67}$ TPSFHEK $^{73}$<br>$^{74}$ SKNGTEVTEYR $^{84}$<br>$^{76}$ NGTEVTEYR $^{84}$<br>$^{87}$ GKPVEIDVK $^{95}$ | 0.537 | - |
| BCAM1242 (HP) | $^{90}$ LQVVALVEGGK $^{100}$ | | $^{37}$ WAGIASAR $^{44}$<br>$^{137}$ AFGVVFTSSAR $^{147}$ | 0.486 | $^{20}$ AADSQPCSDE $^{29}$<br>$^{48}$ RDADR $^{52}$<br>$^{78}$ LPKSKNPDE $^{86}$<br>$^{103}$ AADRSVVEEDVVTEIDE $^{119}$ |

| | | | | | |
|---|---|---|---|---|---|
| | | | | | $^{126}$ TAPYRLSP $^{133}$<br>$^{146}$ **AR**GPSCPDANAS $^{157}$<br>$^{169}$ RIRPV $^{173}$<br>$^{180}$ GWVSVEGDACGPGAGDARSE $^{199}$<br>$^{208}$ EKTSSHG $^{214}$<br>$^{225}$ TKTQRKNGDYSDTGKRTA $^{242}$<br>$^{254}$ GIDMFRNFWYSPTAM $^{268}$ |
| BCAM1576 (Phosphoesterase family protein) | $^{62}$ HIVVIYAENR $^{71}$<br>$^{145}$ITDAQGKPLPNGV ITR$^{160}$<br>$^{489}$ IPALVISPFAK $^{499}$<br>$^{520}$ VHGLAPLDGVVLR $^{532}$<br>$^{533}$ DNAFAAR $^{539}$ | | $^{104}$ TPLPVLPK $^{111}$<br>$^{134}$ DIDKLPNAPFR $^{144}$<br>$^{166}$ FYQNQMQIAGGR$^{177}$<br>$^{256}$ VEGDDPAGTR $^{265}$<br>$^{268}$ LADDSPASALDGPPK $^{282}$ | 0.489 | $^{5}$ PDRPDDLPADPD $^{16}$<br>$^{40}$ AGNAPRSAADLRIDEALRQ $^{58}$<br>$^{147}$ **DAQGKPL** $^{153}$<br>$^{181}$FAAWADSGGLVMGHYRNSAD TLR$^{203}$<br>$^{241}$ PDAHKHPHA $^{249}$<br>$^{256}$ **VEGDDPAG** $^{263}$<br>$^{273}$ **PASALDGPPK**FAVD $^{286}$<br>$^{310}$ PPDPGNAAYADPADHRVMPPQ $^{330}$<br>$^{470}$ GWWDHVAPPVGDRWG $^{484}$<br>$^{500}$ KGFVDHTL $^{507}$ |
| BCAM1761 (Putative lipoprotein) | $^{52}$LSGTEQSQHNGVTDIAVGSN SYFVTLTPSGNGSVIK$^{87}$ | | $^{91}$ GSGSEPAEEAMR $^{102}$ | 0.484 | $^{17}$ GSAPSSDSAGSGAA$^{30}$<br>$^{38}$ QRPAY $^{42}$<br>$^{53}$ **SGTEQSQHNGVTD** $^{65}$<br>$^{90}$ R**GSGSEPAEEAMR** $^{102}$ |
| BCAM1876 (HP) | | | $^{41}$ TFGGLAAQANK $^{51}$<br>$^{41}$ TFGGLAAQANKK $^{52}$<br>$^{59}$ FKDVTAR $^{65}$ | 0.503 | $^{9}$ SASAQQS $^{15}$<br>$^{24}$ QAQLQNNATDQ $^{34}$<br>$^{96}$ GITDESDGIAE $^{106}$ |
| BCAM1920 (HP) | | $^{1}$MAVTIDLGSGK$^{11}$ | $^{13}$ ATLSSDSGSIR $^{23}$ | 0.520 | $^{8}$ **GSGKRATLSSDSGSIR**TAEQRA $^{29}$<br>$^{39}$ PRYPGL $^{44}$<br>$^{56}$ GLAGSLSGD $^{64}$<br>$^{219}$ KDANDLGG $^{226}$ |
| BCAM1921 (Putative phage membrane protein) | | | $^{240}$ NNPALMTDTLDASMR $^{254}$<br>$^{330}$ LGGQVSNDVVYAR $^{342}$ | 0.495 | $^{39}$ GGSVPFGQIDSR $^{50}$<br>$^{67}$ MVEFRPIKTKRG $^{78}$<br>$^{97}$ NAAPSDNE $^{104}$<br>$^{110}$ FQDMRFPPGTDESMC $^{124}$ |

| | | | | |
|---|---|---|---|---|
| | | | | $^{135}$ TEMRN $^{139}$<br>$^{152}$ GRIDV $^{156}$<br>$^{172}$ SNQGRKAWKDPRSAEWS $^{188}$<br>$^{252}$ **SMR**ANMQGAAQRGMD $^{266}$<br>$^{328}$ VK**LGGQVSNDVV** $^{339}$ |
| BCAM1931<br>(Putative porin) | | $^{44}$ SLWSMGSGIDQSR $^{56}$<br>$^{61}$ GSEDLGGGLK $^{70}$<br>$^{96}$ QAFVGLSSQYGTVTLGK $^{112}$<br>$^{144}$ LSTNGDVALNNSIK $^{157}$<br>$^{199}$LGAAYSQANLGDGTNANGATNIAA<br>QGR $^{225}$<br>$^{263}$ ADNYEVNAK $^{271}$<br>$^{312}$ RTDVYAQAVYQR $^{323}$<br>$^{313}$ TDVYAQAVYQR $^{323}$ | 0.513 | $^{36}$ YQSNVASK**SLWSMGSGID** $^{53}$<br>$^{63}$ **EDLGGG** $^{68}$<br>$^{79}$ FNIGNGRLGNNGGMF $^{93}$<br>$^{173}$ SFSNNQNFGNNRA $^{185}$<br>$^{192}$ YQFQG $^{196}$<br>$^{205}$**QANLGDGTNANGATNIAAQ**<br>**GR**V$^{226}$<br>$^{248}$ QSRIDNRAAGVPSLR**ADN** $^{265}$<br>$^{272}$ YNLTPA $^{277}$<br>$^{287}$ TNAKVDGSSAHW $^{298}$<br>$^{323}$ RASKNAGGASIYNGDLSTAPSSSI $^{346}$ |
| BCAM1933<br>(Putative cyclase) | | $^{33}$ DLAAEEANR $^{41}$<br>$^{42}$ QLVLTFYDR $^{50}$<br>$^{55}$ HEAVEAAAVVADDYK $^{69}$<br>$^{124}$ GEAVVDIFR $^{132}$<br>$^{138}$ IVEHWDVIQPVPETSANR $^{155}$ | 0.476 | $^{24}$ ALAAGPATR**DLAAEEANRQ** $^{42}$<br>$^{67}$ **DYK**QHNPHVPDGKQP $^{81}$<br>$^{118}$ ERPGDR**G** $^{124}$<br>$^{142}$ **WDVIQPVPETSANR**N $^{156}$ |
| BCAM2181<br>(HP) | | $^{96}$ KGLAPVEPR $^{104}$<br>$^{150}$ VEALLADESTAAR $^{162}$ | 0.506 | $^{25}$ AGMDEVPRMSNDVYR $^{39}$<br>$^{46}$ SNVTADEKIMCQGDMIA $^{62}$<br>$^{124}$VYESGPASRARHHAALQQKAPD<br>VAP$^{148}$<br>$^{155}$ **ADESTAAR**R $^{163}$<br>$^{178}$ PEASAR $^{183}$ |
| BCAM2418<br>(Trimeric<br>autotransporter<br>adhesin) | | $^{205}$GAQSAASDGIAIGGQSSVASAATSGIA<br>VGR$^{234}$<br>$^{235}$ GATVNGAYGIAQGDGVVSGATGR $^{257}$<br>$^{258}$ NVAIGSSGTTANSATSAGGAVAI<br>GR $^{282}$ | 0.681 | $^{2748}$ TENLKA $^{2753}$<br>$^{2756}$ NGGFSGGQKVIGA $^{2768}$ |
| BCAM2549 | $^{185}$ ADQAQSEALFR $^{195}$<br>$^{214}$ QLDSDQDLYR $^{223}$ | $^{240}$ FNEGDISELDVSR $^{252}$ | 0.521 | $^{4}$ DRKMDNMHNTNGLMR $^{18}$<br>$^{175}$ RVGRNVEASR**ADQAQSEAL** $^{193}$ |

| | | | | | |
|---|---|---|---|---|---|
| (Multidrug efflux system outer membrane protein OpcM) | $^{253}$AKNELASAQADAVGV AR$^{269}$<br>$^{396}$ AQYDEQVANYR $^{406}$<br>$^{415}$ EVEDNLADLR $^{424}$ | | | | $^{264}$ **AVGVAR**RRAA $^{273}$<br>$^{286}$ PADFAFKETPIVPVAVKIPPGL $^{307}$<br>$^{316}$ PDVSA $^{320}$<br>$^{332}$ IGLAKSAYFPK $^{342}$<br>$^{346}$TGSFGYEASTLGNLFLWSSRTFLLG$^{370}$<br>$^{421}$**ADLR**LLDDQIRAQEAAVNASRR AAT$^{445}$<br>$^{448}$ RTQYQEGEVAYL $^{459}$<br>$^{466}$ RSVLQSQLQANQ $^{477}$<br>$^{495}$ GGWGNAPAPTAVGDAASGKAD $^{515}$ |
| BCAM2603 (HP) | $^{19}$YDGLTALNAYDEDGR$^{33}$ | | $^{37}$ YAITEGPYAGAK $^{48}$<br>$^{73}$ ATVVHIDDFAAGTSR $^{87}$ | 0.507 | $^{5}$ TQRPPFAGK $^{13}$<br>$^{21}$ **GLTALNAYDEDGR** $^{33}$<br>$^{40}$ **TEGPYAGAK**GEVE $^{52}$<br>$^{56}$ QRIAGE $^{61}$<br>$^{65}$ ISWQEAD $^{71}$<br>$^{80}$ **DFAAGTSR**S $^{88}$<br>$^{97}$ FYRLDGS $^{103}$ |
| BCAM2686 (HP) | | $^{118}$TVESQAADAEAAQPAVEAA VVK $^{139}$<br>$^{76}$LAAQAADLPAGAGA ATK$^{92}$ | $^{108}$ LQAADGVAAR $^{117}$ | 0.543 | $^{318}$ HLKQDVATKQQRIDR $^{332}$<br>$^{351}$ HEALLSQ $^{357}$<br>$^{359}$ DRLQRNANREAAVAKRV $^{375}$ |
| BCAM2761 (Giant cable pilus - CblA) | | | $^{80}$ LATAPALK $^{87}$<br>$^{80}$ LATAPALKNQTSPGAAEIPLSVK $^{102}$<br>$^{88}$ NQTSPGAAEIPLSVK $^{102}$<br>$^{102}$ LGETELTTTAATLK $^{116}$ | 0.501 | $^{67}$ IFTNDKAKD $^{75}$<br>$^{85}$ **ALKNQTSPGAA** $^{95}$ |
| BCAS0104 (A-type flagellar hook-associated protein 2 - FliD2) | | $^{52}$ VATLAASQASGNTR $^{65}$<br>$^{386}$ DGTLNVDSAK $^{395}$<br>$^{396}$ LDSALSANPSGVAR $^{409}$<br>$^{421}$ LADQITQFTR $^{430}$<br>$^{481}$ MNTNSQYLTR $^{490}$<br>$^{491}$ LFGGANSNGTLSK $^{503}$ | $^{439}$ TTALNADLK $^{447}$<br>$^{449}$ VAQQQSDLSDYAAQLTK $^{465}$ | 0.517 | $^{57}$ **ASQASGNT** $^{64}$<br>$^{84}$ TSLKNGALQSTFNAVASG $^{101}$<br>$^{129}$ ALSSSGFNGSKALGT $^{143}$<br>$^{201}$ KTGSANAI $^{208}$<br>$^{302}$ DTKSQAA $^{308}$<br>$^{326}$ ALSSYTAGASSQGALIGDST $^{345}$<br>$^{358}$ ARGVDGGGATEKGNGHVN $^{375}$<br>$^{483}$ **TNSQYLTR** $^{490}$<br>$^{494}$ **GANSNGTL** $^{501}$ |

| | | | | | |
|---|---|---|---|---|---|
| BCAS0151 (HP) | | $^{11}$SPLADSGVTANAPAATSSSGG TTDAASTTGTTGATGATDTTR$^{44}$ $^{58}$DSTGATGETLGAAGASSEDPA VAQLK$^{83}$ | $^{1}$ MQIDFSTTAR $^{10}$ | 0.567 | $^{94}$ ATLERQ $^{99}$<br>$^{104}$ AQRAKDDPAAAVEQQSLSA $^{122}$<br>$^{144}$ KSGGSPSGGLV $^{154}$ |
| BCAS0236 (Trimeric autotransporter adhesin) | $^{1057}$QITNVADGTGQQDAVT VR$^{1074}$ | | $^{1017}$ AVVPSSGILR $^{1026}$ | 0.667 | - |
| BCAS0522 (HP) | | | $^{3}$ THQPILTTSVTAAAGLNR $^{20}$<br>$^{85}$ ATGASNGYALDAATAAGDVIR $^{105}$ | 0.501 | $^{19}$ **NR**FQFVGFDGGV $^{30}$<br>$^{43}$ TTADVGEQA $^{51}$ |
| BCAS0750 (HP) | | $^{171}$ GSASAQGGAL $^{180}$ | $^{130}$ SHAQGAVSTAGDVAGGAK $^{147}$<br>$^{150}$ AGEALEGATNAVQGGASVGVK $^{170}$ | 0.568 | - |

[1] Peptides identified using surface-shaving with trypsin and LC-MS/MS analysis.

[2] http://tools.iedb.org/bcell/. Threshold used of 0.5.

[3] Epitopes shorter than 5 or larger than 25 amino acids were not considered. At bold are the predicted B cell epitopes found by the Surface-shaving approach.

[*] Peptide has an oxidation in the fourth amino acid (Methionine)
